# Effects of aerobic, resistance and combined training on endothelial function and arterial stiffness in older adults: A systematic review and meta-analysis

**DOI:** 10.1371/journal.pone.0308600

**Published:** 2024-12-02

**Authors:** Raphael Silveira Nunes da Silva, Diego Silveira da Silva, Patrícia Caetano de Oliveira, Gustavo Waclawovsky, Maximiliano Isoppo Schaun

**Affiliations:** Instituto de Cardiologia do Rio Grande do Sul/Fundação Universitária de Cardiologia, Porto Alegre, Brasil; Università degli Studi di Milano: Universita degli Studi di Milano, ITALY

## Abstract

We conducted a systematic review of randomized clinical trials evaluating the effects of aerobic, resistance and/or combined training on flow-mediated dilation (FMD) and/or pulse wave velocity (PWV) in older adults. The studies were selected from the electronic databases PubMed, Cochrane, LILACS, EMBASE, Web of Science, and the gray literature. We assessed the studies using Cochrane risk of bias (RoB2) tool and the GRADE tool. The GRADE assessment showed moderate quality of evidence for aerobic training and resistance training and very low for combined training. The measures of effects are presented as mean differences of the intervention group versus the control group and related 95% confidence intervals (95% CIs) pooled by a random-effects model using an inverse variance method. Our analysis of 24 RCTs (Intervention group [n = 251]: 67.7 ± 5.6 years old; control group [n = 228]: 68.7 ± 5.9 years old) showed that aerobic training was effective to improve FMD (0.64% [95% CI 0.24 to 1.03], p = 0.002) and PWV (–1.21 m/s [95% CI –1.37 to –1.05], p< 0.001) by compared to the control group. The subgroup analyses showed no FMD differences following aerobic training in healthy adults when compared to those with any health condition. Combined training was effective in improving FMD (0.60% [95% CI 0.50 to 0.71], p< 0.001) and PWV (-0.79 m/s [95% CI –1.23 to –0.35], p = 0.002). But these same parameters did not show any improvement in response to resistance training. A major limitation of this study is that the analysis to evaluate the effect of resistance training on PWV include only one study, and no inferences could be made from the data. Aerobic and combined training, but not resistant training, improve flow-mediated dilation and pulse wave velocity in the elderly. PROSPERO: CRD42021275282.

## Introduction

The number of people aged 60 or over will increase to 1.4 billion by 2030 and 2.1 billion by 2050 according to global estimates [1]. This fact deserves attention as aging is a significant non-modifiable risk factor for cardiovascular diseases (CVDs) [2]. In addition, physical inactivity is a major contributing factor for cardiovascular events [3]. Physically active individuals have up to 35% less risk of mortality from CVDs [3] and the risk of death from all causes in older adults with high levels of physical activity can be reduced by up to 45% [4]. However, nearly 45% of adults aged ≥60 years do not meet the minimum recommended amount of physical activity [5]. In the United States (USA), managing health conditions due to physical inactivity has been estimated to cost the health system over 100 billion dollars a year [6].

Vascular dysfunction is characterized by reduced endothelial function and increased arterial stiffness^7^. Aging and physical inactivity are strongly associated with vascular dysfunction further increasing the risk of developing CVDs [7]. In this manner, Flow-mediated dilation (FMD) and pulse wave velocity (PWV) are valuable methods to assess endothelial function and arterial stiffness. Several meta-analyses have shown that every 1% increase in FMD is associated with a lower risk (8–16%) of fatal and non-fatal cardiovascular events and/or deaths from all causes with even greater effects in individuals with established CVDs [8]. Additionally, a 1m/s reduction in PWV has been associated with reduced risk of cardiovascular events (12–14%), CVD death (13–15%) and deaths from all causes (6–15%) [9]. Some studies have showed that different modalities of training (aerobic, resistance and/or combined) produced FMD improvements [10] while others have reported no effect [11, 12]. As for PWV, studies of resistance training have evidenced an association with increased arterial stiffness in healthy young individuals [13]. In turn, one meta-analysis has shown that resistance training has no effect on PWV [14]. In addition, we found other meta-analyses that evaluated the effect of other proposed training modalities on PWV in participants aged 7 to 78 years including sub-analyses of young, middle-aged and older adults as a single group [15]. Other meta-analyses reported results for populations with highly heterogeneous health status [16, 17] and metabolic or hemodynamic conditions (e.g., diabetes and hypertension) regardless of age [18, 19].

Bearing in mind that there is no consensus in the literature on the effects of exercise training on FMD and PWV in adults aged ≥60 years, we conducted a systematic review and meta-analysis of RCTs to examine the effects of aerobic, resistance and combined exercise training on FMD and PWV in older adults.

## Methods

### Protocol and registration

Our review followed the guidelines of the *Preferred Report Items for Systematic Reviews and Meta-Analysis* (PRISMA). We used the *Population*, *Intervention*, *Comparison*, *Outcomes and Study* (PICOS) framework and the methodology described in the *Cochrane Handbook for Systematic Reviews of Interventions* [20]. The protocol of this systematic review and meta-analysis was registered in the *International Prospective Register of Systematic Review* (PROSPERO) (CRD42021275282, registered on September 29, 2021). The database used in this study is available at Mendley Data (https://data.mendeley.com/datasets/chkhyxp43p/2; Published: 11 Jan 2024; DOI:10.17632/chkhyxp43p.2). A more detailed description of the methodology can be found elsewhere [21] (S1 Chart in S1 File).

### Search strategy

We conducted searches in the electronic databases Medline (PubMed), Embase, Cochrane, Web of Science, LILACS, OpenGrey, the Brazilian Coordination for the Improvement of Higher Education Personnel (CAPES) bank of theses and dissertations, the Brazilian Clinical Trials Registry (ReBEC), Clinical Trial.gov and WHO International Clinical Trials Registry Platform (WHO–ICTRP). We also searched for meta-analyses of similar subjects and references cited in eligible RCTs. Searches were carried out up to March 2024 using the following MeSH terms: “exercise,” “vascular endothelium,” and “vascular stiffness.” To increase accuracy and sensitivity of our searches, the terms for RCTs in the Medline and EMBASE databases were added to the search terms [17, 18]. The search was not limited by language and date of publication. Search terms are described in the S2 Chart in S1 File.

### Study selection

Two reviewers (RSNS and DSS) independently screened the articles based on their titles and abstracts using EndNote X9–BLD12062. Our review was based on the PICOS framework and pre-specified eligibility criteria [21] as summarized below.

### Inclusion criteria

- Design: RCT.
- Population: individuals aged ≥ 60 years (main population);
- Intervention: aerobic, resistance and combined (aerobic + resistance) exercise training;
- Comparator: (no exercise) control;
- Intervention duration: ≥ 4 weeks;
- Exercise frequency: ≥ 2 times per week;
- Outcome: endothelial function assessed by FMD and vascular stiffness assessed by PWV;

### Exclusion criteria

- Intervention: alternative forms of exercise (including martial arts, exercise for relaxation, yoga, and muscle electrostimulation); dietary interventions; nutritional supplements; and medication use.
- Outcome: cellular, biochemical and other outcomes assessing endothelial function and arterial stiffness by methods other than FMD or PWV.
- Duplicate publications and same participant sample used in two or more trials (data from only one of these studies were included in the analysis);
- Design: cohort, observational, and case-control studies; case reports; reviews; and protocols.

Our reviewers (RSNS and DSS) read all potentially eligible studies in full text and selected those that met all inclusion criteria for data extraction. The authors were contacted by email (three attempts) to obtain any additional information if needed. Any disagreements were resolved by a third reviewer (GW).

### Data extraction and management

Our reviewers (RSNS and DSS) manually extracted and compiled the data in a spreadsheet (Microsoft Excel 365 for Windows). For outcomes of interest in graphs we used WebPlotDigitizer to extract data. We extracted data from all participants in each group, including sample sizes, control and pre- and post-intervention FMD and PWV mean values, and related measures of dispersion (standard error [SE], standard deviation [SD], and confidence interval [CI]). We also extracted mean differences (▲ = post-intervention–pre-intervention) and related measures of dispersion when available, FITT information (frequency, intensity, time and type of exercise), sample description data and methods of FMD and PWV assessment.

For studies measuring arterial stiffness at different arteries, we chose to extract data for central PWV measurements (carotid-femoral or carotid-brachial).

### Risk of bias and strength of evidence

The risk of bias of eligible studies was assessed using Cochrane Risk of Bias 2 (RoB2) tool [22]. This assessment is based on a set of six domains and classified as low risk of bias, some concerns or high risk of bias as described elsewhere [21]. Since it is difficult to blind participants in exercise training studies, all studies were judged as some concerns of bias in the domain “deviations from intervention.”

The strength of the body of evidence was assessed using the *Grading of Recommendations*, *Assessment*, *Development and Evaluation* (GRADE) tool [20]. The GRADE tool classifies the certainty of evidence into four levels (high, moderate, low and very low) based on the assessment of confidence in specific estimates in five domains: methodological limitations (risk of bias); inconsistency; indirectness of evidence; imprecision; and publication bias [21].

### Statistical analysis

The measures of effects are presented as mean differences (MDs) of the intervention group versus the control group and related 95% confidence intervals (95% CIs) pooled by a random-effects model using an inverse variance method. We considered the calculated values for a prediction interval (PI) as they reflect the interval of uncertainty of the effects to be expected in future RCTs [23].

Heterogeneity was assessed using the DerSimonian and Laird method (*tau*^2^) and relative variability by the Higgins inconsistency test (*I*^*2*^) [21, 24]. To explore the heterogeneity of the studies, we conducted subgroup analyses and/or meta-regression for potential effect modifiers (including body mass index [BMI], baseline FMD, baseline PWV and FITT components) [20, 21]. For subgroup analyses, we determine: 1) "healthy individuals and sick individuals." It was adopted as "healthy" in all studies that described Fits population as showing no determining factor for changes in the cardiovascular state beyond age; 2) "resistant or dynamic training," as described by the authors; 3) "evaluation site for the PWV, as explained by each author. Forest plots were constructed to visualize the effect estimate of individual studies based on non-CI overlapping that is due to heterogeneity [20, 24]. We performed the Egger’s test using a funnel plot to assess potential publication bias when applicable (≥10 studies). To avoid unit-of-analysis errors for RCTs with multiple treatment arms and a single control group, the number of participants in the control group was weighted by the number of groups and participants undergoing the intervention [11]. When the change in SD was not reported, it was imputed by using a value of 0.5 for the correlation coefficient (CC) [25]. The CC was calculated according to section 6.5.2.8 of the Cochrane Handbook for Systematic Reviews of Interventions [20]: Δ SD = √ SD^2^_baseline_ + SD^2^_end_−(2 * CC * SD _baseline_ * SD _end_). All measures of dispersion presented as SEs or CIs were converted into SDs before the meta-analysis. All statistical tests were two-tailed and the significance level was set at p<0.05. Data modelizations were performed with RStudio (version 1.3.959) using the r package “meta” for Windows (version 3.6.1). A RStudio script was written to guide the meta-analysis (S2 Chart in S1 File).

## Results

**Fig 1:**
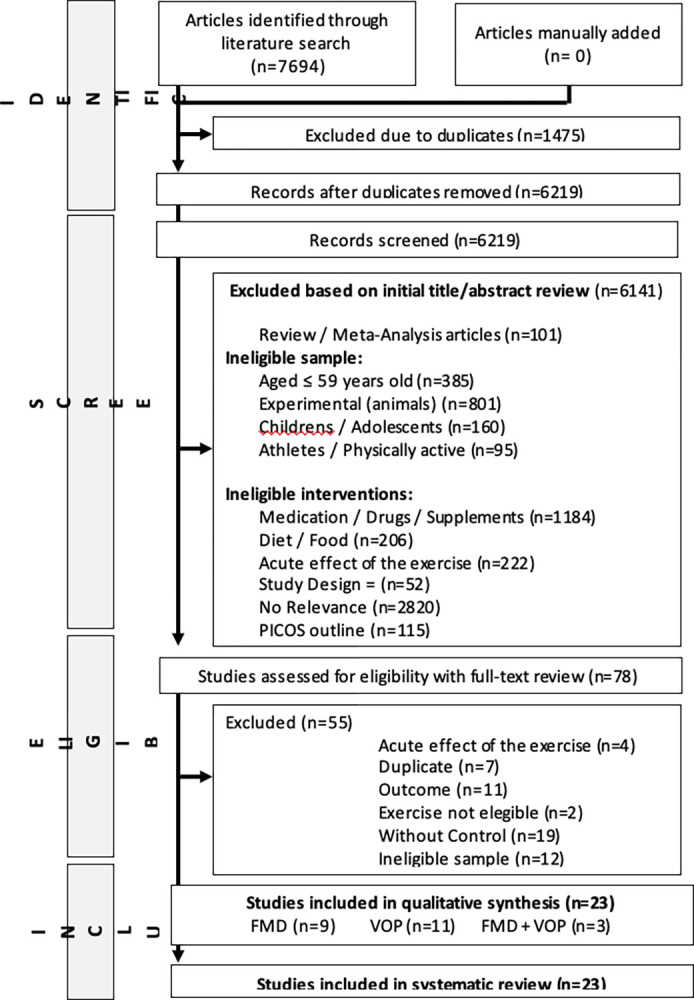
Flowchart of the selection of randomized controlled trials for the systematic review and meta-analysis.

Fig 1 summarizes the flowchart for the selection of the studies. In brief, 10,086 studies were retrieved in our initial search, of which 1,696 were duplicates. We read the titles and abstracts of the remaining 8,390 studies and screened out 8,307 based on the PICOS framework. We then read full text of 83 potentially eligible studies; 59 were excluded as they did not meet the eligibility criteria. Thus, 24 studies were selected for the systematic review and meta-analysis including nine studies assessing FMD, 12 assessing PWV and three assessing both.

A total of 6,810 articles were retrieved from the gray literature. After screening through titles and abstracts, eight studies remained which were further screened for full text. We excluded five due to ineligible sample (aged ≤ 59 years) and three other studies retrieved from ClinicalTrials.gov database reported incomplete results (studies at a very early stage or undergoing recruitment).

### Description of studies assessing FMD

Table 1 presents a general description of the studies assessing FMD. The final analysis included 479 participants—251 in the intervention group and 228 in the control group. Mean age was 67.7 ± 5.6 years in the intervention group and 68.7 ± 5.9 years in the control group. There was a total of 311 women and 168 men; two studies involved women only [26, 27] and one involved men only [28]. As for clinical characteristics, five studies evaluated healthy adults [26, 28–31] and seven adults with comorbidities (including systemic arterial hypertension [32, 34] heart failure with preserved ejection fraction [35], depression [27], and diabetes mellitus [36, 37]). As for the FITT components, the average frequency of exercise was three times a week (range 2–7 times a week) and the average duration of the intervention was 12 weeks (range 8–24 weeks). Of the studies involving aerobic exercise training, exercise intensity was calculated from heart rate reserve (HRR) in five studies (range 40–70%; mean 55%) [27, 28, 30, 34, 35]. Two studies used maximum heart rate (HRmax) (range 60–80%; mean 71%) [31, 37], two used lactate threshold (range 2–2.5mmol/L) [32, 33] and one study used ventilatory threshold [29]. Of those studies involving resistance training, three used loads from one-repetition maximum test (1-RM) [26, 28, 37], one used the OMNI-Resistance Exercise Scale [36] and one used the maximum voluntary isometric contraction (CVM) [34]. Exercise sessions lasted on average 42 minutes (range 30–60 minutes). This variable was not available in one study, and it was not included in the average calculation described here [36]. As for exercise modality, eight studies evaluated the effects of aerobic training [27, 29–35], three the effects of resistance training [26, 34, 36] and two the effects of combined training [28, 37]. Data were extracted from graphs using WebPlotDigitizer for three studies [28, 31, 36] and data from three other studies were converted from SE to SD [26, 27, 31].

**Table 1:** Description of the studies selected (assessing FMD).

Reference	Intervention	Health status	*n*	Gender (%)	Mean age ± SD (years)	Mean BMI (kg/m^2^)	Intervention protocol	% FMD (protocol)	% FMD (result)
**Bouaziz et al. 2019 [29]**	**Aerobic** (interval)	Healthy	TG: 30 CG: 30	M: 21.7 F: 78.3	TG: 74.3 ± 3.4 CG: 72.9 ± 2.5	TG: 28.7 CG: 28.8	Frequency: 2 times/wk Training duration: 9.5 wks Intensity: 6 sets (4 min VT1 + 1 min 40% VT1) Session duration: 30 min	Occlusion time: 5 min Cuff location: forearm Measuring site: arm Cuff pressure: 200 mmHg	ΔGT: + 0.8 ± 0.7* ΔCG: - 0.4 ± 0.2
**Haynes et al. 2020 [30]**	**Aerobic** (continuous—swimming pool)	Healthy	TG: 18 CG: 16	M: 21.2 F: 78.8	TG: 62.2 ± 7.4 CG: 61.8 ± 7.3	TG: 26.8 CG: 27.4	Frequency: 3 times/wk Training duration: 24 wks Intensity: 55–65% HRR Session duration: 50 min	Occlusion time: 5 min Cuff location: forearm Measuring site: arm Cuff pressure: 220 mmHg	ΔGT: + 0.5 ± 0.0 ΔCG: - 0.1 ± 0.1
**Jaime et al. 2019 [26]**	**Resistance**	Post-menopausal women	TG: 12 CG: 08	M: 0 F: 100	TG: 64.0 ± 3.4 CG: 67.0 ± 2.8	TG: 24.0 CG: 22.5	Frequency: 2 times/wk Training duration: 12 wks Intensity: 2–3 sets 15 reps at 40% (1-RM) Session duration: 30–35 min	Occlusion time: 5 min Cuff location: forearm Measuring site: arm Cuff pressure: 50 mmHg above SBPmax	ΔGT: + 5.0 ± 0.3** ΔCG: - 2.0 ± 0.8
**Kitzman et al. 2013 [35]**	**Aerobic** (continuous)	HFpEF	TG: 32 CG: 31	M: 21.2 F: 78.8	TG: 70.0 ± 7 CG: 70.0 ± 7	TG: 23.8 CG: 76.2	Frequency: 3 times/wk Training duration: 16 Intensity: 70% HRR Session duration: 60 min	Occlusion time: 4 min Cuff location: forearm Measuring site: arm Cuff pressure: 50 mmHg above SBPmax.	ΔGT: - 0.2 ± 1.0 ΔCG: - 0.4 ± 0.0
**Pierce et al. 2011 [31]**	**Aerobic** (continuous)	Healthy	TG: 26 CG: 10	M: 41.67 F: 58.33	TG: 63 ± 5.1 CG: 60 ± 3.2	TG: 25.3 CG: 25.1	Frequency: 6-7times/wk Training duration: 8 wks Intensity: 70–75% HRmax Session duration: 40–50 min	Occlusion time: 4 min Cuff location: forearm Measuring site: arm Cuff pressure: 250 mmHg	ΔGT: + 1.3 ± 0.8 ΔCG: + 0.0 ± 0.5
**Prakhinkit et al. 2014 [27]**	**Aerobic** (continuous)	Depression	TG: 13 CG: 13	M: 0 F: 100	TG: 74.8 ± 6.1 CG: 81.0 ± 6.1	TG: 26.0 CG: 24.0	Frequency: 3 times/wk Training duration: 12 wks Intensity: 40–50% HRR Session duration: 30 min	Occlusion time: 5 min Cuff location: forearm Measuring site: arm Cuff pressure: 50 mmHg above SBPmax	ΔGT: + 3.8 ± 0.3* ΔCG: - 0.3 ± 3.2
**Rech et al. 2019 [36]**	**Resistance**	Diabetes (type 2)	TG: 17 CG: 21	M: 52.6 F: 47.4	TG: 70.5 ± 7.4 CG: 68.0 ± 6.5	TG: 28.4 CG: 28.3	Frequency: 3 times/wk Training duration: 12 wks Intensity: 10–12 reps submax (15 RM) Session duration: NA	Occlusion time: 5min Cuff location: forearm Measuring site: arm Cuff pressure: 240 mmHg	ΔGT: + 1.0 ± 0.2 ΔCG: + 0.5 ± 0.2
**Scheer et al. 2019 [37]**	**Combined** (circuit—swimming pool)	Diabetes (type 2)	TG: 13 CG: 14	M: 55.6 F: 44.4	TG: 60.9 ± 9.6 CG: 63.9 ± 9.8	TG: 35.3 CG: 29.5	Frequency: 3 times/wk Training duration: 12 Intensity: 60–80% HRmax and Borg scale score 12–15 Session duration: 50 min	Occlusion time: 5 min Cuff location: forearm Measuring site: arm Cuff pressure: 220 mmHg	ΔGT: + 0.4 ± 0.6 ΔCG: - 0.8 ± 0.0
**Shiotsu et al. 2018 [28]**	**Combined** (individual)	Healthy	TG: 15 CG: 10	M: 100 F: 0	TG: 70.4 ± 4.1 CG: 71.0 ± 4.4	TG: 23.6 CG: 23.8	Frequency: 2 times/wk Training duration: 10 Intensity: 60% HRR + 3 sets of 8–12 reps (60–70% 1-RM). Session duration: 40 min	Occlusion time: 5 min Cuff location: forearm Measuring site: arm Cuff pressure: 200 mmHg	ΔGT: + 0.2 ± 0.0 ΔCG: - 0.4 ± 0.1
**Westhoff et al. 2007 [32]**	**Aerobic** (continuous)	Hypertension	TG: 27 CG: 27	M: 48.2 F: 51.8	TG: 67.2 ± 4.8 CG: 68.9 ± 5.2	TG: 27.7 CG: 29.8	Frequency: 3 times/wk Training duration: 12 Intensity: lactate 2.5mmol/L Session duration: 36 min	Occlusion time: 3 min Cuff location: forearm Measuring site: arm Cuff pressure: 300 mmHg	ΔGT: + 0.2 ± 0.2 ΔCG: - 0.8 ± 1.8
**Westhoff et al. 2008 [33]**	**Aerobic** (continuous)	Hypertension	TG: 12 CG: 12	M: 45,8 F: 54,2	TG: 66,1 ± 4,0 CG: 68,4 ± 9,7	TG: 28,6 CG: 26,5	Frequency: 3 times/wk Training duration: 12 wks Intensity: lactate 2.0 mmol Session duration: 30 min	Occlusion time: 3 min Cuff location: forearm Measuring site: arm Cuff pressure: 300 mmHg	ΔGT: + 0,3 ± 0,3 ΔCG: 0,0 ± 0,4
**Yoon et al. 2019 a [34]**	**Resistance** (isometric handgrip)	Hypertension	TG: 17 CG: 18	M: 25,7 F: 74,3	TG: 67,0 ± 5,0 CG: 70,0 ± 6,0	TG: 26,5 CG: 26,7	Frequency: 3 times/wk Training duration: 12 wks Intensity: 30% MVC Session duration: 2 sets x 2 min (each arm)	Occlusion time: 5 min Cuff location: forearm Measuring site: arm Cuff pressure: 250 mmHg	ΔGT: + 0,6 ± 0,2 ΔCG: + 0,3 ± 0,4
**Yoon et al. 2019 b [34]**	**Aerobic** (continuous)	Hypertension	TG: 19 CG: 18	M: 27,0 F: 73,0	TG: 70,0 ± 6,0 CG: 70,0 ± 6,0	TG: 26,4 CG: 26,7	Frequency: 3 times/wk Training duration: 12 wks Intensity: 40–60% HRR Session duration: 30 min	Occlusion time: 5 min Cuff location: forearm Measuring site: arm Cuff pressure: 250 mmHg	ΔGT: + 0,5 ± 0,3 ΔCG: + 0,3 ± 0,4

HFpEF, heart failure with preserved ejection fraction; 1-RM, one-repetition maximum; MVC, maximal voluntary contraction; HRmax, maximum heart rate; HRR, heart rate reserve; CG, control group; GT, training group; VT1, first ventilatory threshold; min, minutes; mmHg, millimeter of mercury; NA, not available; wks: weeks; M, males; F, females

*p<0.05

**p<0.01.

### Description of studies assessing PWV

Table 2 presents a general description of the studies assessing PWV. The final analysis involved a total of 778 participants—406 in the intervention group and 372 in the control group. Mean age was 68.6 ± 3.3 years in the intervention group and 68.3 ± 4.1 years in the control group. There was a total of 568 women and 209 men; six studies evaluated women only [38–42], two evaluated men only [28, 43], and one evaluated both men and women [44]. One study that did not provide information on the proportion of men and women in the groups and was not included in this analysis [45]. Ten studies evaluated healthy adults [28, 29, 38–41, 44, 46–48] and seven adults with comorbidities (including Alzheimer’s disease [36], metabolic syndrome [45, 49], systemic arterial hypertension [34, 41, 42], and obesity [43]). As for the FITT components, the frequency of exercise ranged from two to four times a week (mean 2.5 times a week) and duration of the intervention ranged from six to 24 weeks (mean 12 weeks). Exercise intensity was calculated in one study from peak heart rate (PHR) [46], HRR (range 40–85%; mean 60%) in eight studies [28, 34, 38, 39, 42, 45, 48, 49] and HRmax (range 60–70%; mean 68%) in four studies [40, 41, 43, 47]. As for exercise intensity set for resistance training, the OMNI-Resistance Exercise Scale was used in two studies [42, 43], HRmax in another two [40, 41], 1-RM test in one study [28], perceived exertion (borg scale) in one [38], the number of maximum repetitions in one study [44] and MVC in another one [34]. Exercise sessions lasted on average 35 minutes (range 30–90 minutes). Ten studies [29, 34, 37–39, 45–49] evaluated the effects of aerobic training, one the effects of isometric handgrip resistance training [34], one the effects of dynamic resistance training [38] and six the effects of combined training [28, 40–44]. Data were extracted from graphs using WebPlotDigitizer in four studies [28, 39, 42, 47], it was converted from SE to SD in five studies [42, 45–47, 49] and from CI to SD in one study [44]. Finally, eight studies assessed arterial stiffness by carotid-femoral PWV (cfPWV) [28, 29, 34, 44–46, 48, 49], six by brachial-tibial PWV (btPWV) [38, 40–43, 47] and one by carotid-brachial PWV (cbPWV) [39].

**Table 2:** Description of studies selected (assessing PWV).

Reference	Intervention	Health status	*n*	Gender (%)	Mean age ± SD (years)	Mean BMI (kg/m^2^)	Intervention protocol	PWV (artery site evaluated)	PWV (m/s)
**Bouaziz et al. 2019 [29]**	**Aerobic** (interval)	Healthy	TG: 30 CG: 30	M: 21.7 F: 78.3	TG: 74.3 ± 3.4 CG: 72.9 ± 2.5	TG: 28.7 CG: 28.8	Frequency: 2 times/wk Training duration: 9.5 wks Intensity: 6 sets (4min VT1 + 1min 40% VT1) Session duration: 30 min	Carotid-femoral	ΔGT: - 0.7 ± 0.2* ΔCG: - 0.2 ± 0.0
**Kim et al. 2017 a [46]**	**Aerobic** (HIIT)	Healthy	TG: 17 CG: 14	M: 25.8 F: 74.2	TG: 65.0 ± 3.7 CG: 63.0 ± 6.6	TG: 28.1 CG: 25.3	Frequency: 4 times/wk Training duration: 8 wks Intensity: 4 sets of 4min (90% HRpeak) / 3 sets of 3min (70% HRpeak). Session duration: 40 min	Carotid-femoral	ΔGT: 0.0 ± 0.3 ΔCG: + 0.5 ± 0.1
**Kim et al. 2017 b [46]**	**Aerobic** (continuous)	Healthy	TG: 18 CG: 14	M: 37.5 F: 62.5	TG: 65.0 ± 7.2 CG: 63.0 ± 6.6	TG: 28.7 CG: 25.3	Frequency: 4 times/wk Training duration: 8 wks Intensity: 70% HRpeak Session duration: 47 min	Carotid-femoral	ΔTG: -0.5 ± 0.1* ΔCG: + 0.5 ± 0.1
**Kim et al. 2018 a [39]**	**Aerobic** (land)	Healthy	TG: 14 CG: 12	M: 0 F: 100	TG: 67.4 ± 1.8 CG: 66.4 ± 4.5	TG: 24.9 CG: 25.2	Frequency: 2 times/wk Training duration: 16 wks Intensity: 60–70% HRR Session duration: 40 min	Carotid-brachial	ΔTG: - 1.0 ± 0.1** ΔCG: + 0.1 ± 0.1
**Kim et al. 2018 b [39]**	**Aerobic** (swimming pool)	Healthy	TG: 14 CG: 12	M: 0 F: 100	TG: 66.8 ± 3.1 CG: 66.4 ± 4.5	TG: 24.7 CG: 25.2	Frequency: 2 times/wk Training duration: 16 wks Intensity: 60–70% HRR. Session duration: 40 min	Carotid-brachial	ΔTG: -1.2 ± 0.8** ΔCG: + 0.1 ± 0.1
**Kim et al. 2023 [38]**	**Aerobic** (functional circuit)	Healthy	TG: 14 CG: 14	M: 0 F: 100	TG: 68.4 ± 3.5 CG: 67.5 ± 5.1	TG: 24.4 CG: 25.0	Frequency: 3 times/wk Training duration: 16 wks Intensity: 40–60% HRR Session duration: 40 min	Brachial-ankle	ΔTG: -2.4 ± 1.2* ΔCG: + 0.4 ± 0.4
**Kirk et al. 2021 [44]**	**Combined** (resistance + functional circuit)	Healthy	TG: 24 CG: 31	M: 50.9 F: 49.1	TG: 66.6 ± 3.9 CG: 68.2 ± 5.9	TG: 28.1 CG: 26.2	Frequency: 2 times/wk (resistance) + once/wk (functional circuit) Training duration: 16 wks Resistance: 2 sets of 12 reps max, 3-min interval between sets and exercise type Session duration: 40 min Functional circuit: 3 sets of 1 min at 12 exercise stations, 3-min interval per circuit, Intensity: Borg scale score 7–10 (CR-10) Session duration: NA	Carotid-femoral	ΔTG: 1.0 ± 0.3 ΔCG: 1.1 ± 0.3
**Madden et al. 2009 [45]**	**Aerobic** (continuous)	Metabolic syndrome	TG: 17 CG: 17	M: NA F: NA	TG: 71.1 ± 4.9 CG: 71.1 ± 4.0	TG: 30.0 CG: 27.7	Frequency: 3 times/wk Training duration: 12 wks Intensity: 60–75% HRR Session duration: 40 min	Carotid-femoral	ΔTG: -1.8 ± 0.2* ΔCG: + 0.5 ± 0.1
**Madden et al. 2013 [49]**	**Aerobic** (continuous)	Metabolic syndrome	TG: 25 CG: 27	M: 57.7 F: 42.3	TG: 68.5 ± 4.5 CG: 70.0 ± 4.2	TG: 31.6 CG: 28.3	Frequency: 3 times/wk Training duration: 12 wks Intensity: 60–75% HRR Session duration: 40 min	Carotid-femoral	ΔTG: - 3.1 ± 0.3* ΔCG: -1.6 ± 1.1
**Miura et al. 2008 [40]**	**Combined** (circuit)	Healthy	TG: 25 CG: 23	M: 0 F: 100	TG: 69.5 ± 7.0 CG: 68.9 ± 7.5	TG: 23.5 CG: 23.7	Frequency: 2 times/wk Training duration: 12 wks Intensity: ~70% HRmax + 15–20 reps at 6–8 stations (3–5 sets) Session duration: 40 min	Brachial-tibial	ΔTG: - 1.3 ± 0.2 ΔCG: - 0.1 ± 0.2
**Otsuki et al. 2019 [47]**	**Aerobic** (continuous)	Healthy	TG: 23 CG: 26	M: 40.8 F: 59.2	TG: 67.0 ± 8.0 CG: 65.0 ± 7.0	TG: 22 CG: 22	Frequency: once/wk Training duration: 6 wks Intensity: 75% HRmax Session duration: 50 min	Brachial-tibial	ΔTG: - 0.5 ± 0.1* ΔCG: + 0.1 ± 0.1
**Oudegeest-Sander et al. 2013 [48]**	**Aerobic** (continuous)	Healthy	TG: 11 CG: 11	M: 50 F: 50	TG: 68.0 ± 3.0 CG: 71.0 ± 5.0	TG: 27 CG: 24.3	Frequency: 3 times/wk Training duration: 12 wks Intensity: 70–85% HRR Session duration: 30 min	Carotid-femoral	ΔTG: + 0.4 ± 0.6 ΔCG: + 1.2 ± 0.9
**Park et al. 2020 [43]**	**Combined** (alone)	Obesity	TG: 10 CG: 10	M: 100 F: 0	TG: 69.10 ± 0.90 CG: 68.50 ± 0.90	TG: 26 CG: 26.2	Frequency: 3 times/wk Training duration: 12 wks Intensity: (60–70% HRmax) + (3 sets 10–15 reps at OMNI scale score 6–7 [elastic band] Session duration: 90 min	Brachial-tibial	ΔTG: - 0.1 ± 0.0** ΔCG: + 0.1 ± 0.0
**Shiotsu et al. 2018 a [28]**	**Combined** (resistance + aerobic)	Healthy	TG: 15 CG: 10	M: 100 F: 0	TG: 70.4 ± 4.1 CG: 71.0 ± 4.4	TG: 23.6 CG: 23.8	Frequency: 2 times/wk Training duration: 10 wks Intensity: (60% HRR) + (3 sets of 8–12 reps [70–80% 1-RM]) Session duration: 40 min	Carotid-femoral	ΔTG: + 0.1 ± 0.2 ΔCG: + 0.5 ± 0.1
**Shiotsu et al. 2018 b [28]**	**Combined** (resistance + aerobic)	Healthy	TG: 15 CG: 10	M: 100 F: 0	TG: 69.6 ± 4.6 CG: 71.0 ± 4.4	TG: 24.1 CG: 23.8	Frequency: 2 times/wk Training duration: 10 wks Intensity: (60% HRR) + (3 sets of 8–12 reps [70–80% 1-RM]) Session duration: 40 min	Carotid-femoral	ΔTG: - 1.1 ± 0.1* ΔCG: + 0.5 ± 0.1
**Son et al. 2017 [42]**	**Combined** (resistance + aerobic)	Healthy	TG: 10 CG: 10	M: 0 F: 100	TG: 76.0 ± 15.8 CG: 74.7 ± 6.6	TG: 24.05 CG: 22.77	Frequency: 3 times/wk Training duration: 12 wks Intensity: 60%-70% HRR and Borg scale score 11–16 Session duration: 70 min	Brachial-tibial	ΔTG: - 0.8 ± 0.1* ΔCG: + 1.2 ± 0.4
**Yoon et al. 2019 a [34]**	**Resistance** (Isometric handgrip)	Hypertension	TG: 17 CG: 18	M: 25.7 F: 74.3	TG: 67.0 ± 5.0 CG: 70.0 ± 6.0	TG: 26.5 CG: 26.7	Frequency: 3 times/wk Training duration: 12 wks Intensity: 30% MVC Session duration: 2 sets of 2 min per arm (total: 8 min)	Carotid-femoral	ΔTG: - 1.0 ± 0.2** ΔCG: + 0.1 ± 0.1
**Yoon et al. 2019 b [34]**	**Aerobic** (continuous)	Hypertension	TG: 19 CG: 18	M: 27.03 F: 72.97	TG: 70.0 ± 6.0 CG: 70.0 ± 6.0	TG: 26.4 CG: 26.7	Frequency: 3 times/wk Training duration: 12 wks Intensity: 40–60% HRR Session duration: 30 min	Carotid-femoral	ΔTG: -1.1 ± 0.4** ΔCG: + 0.1 ± 0.1

HIIT, high-intensity interval training; 1-RM, one-repetition maximum; MVC, maximal voluntary contraction; HRmax, maximum heart rate; HRR, heart rate reserve; CG, control group; GT, training group; VT1, first ventilatory threshold; min, minutes; mmHg, millimeter of mercury; NA, not available; wks: weeks; M, males; F, females

*p<0.05

**p<0.01.

### Assessment of risk of bias and quality of studies

**Fig 2:**
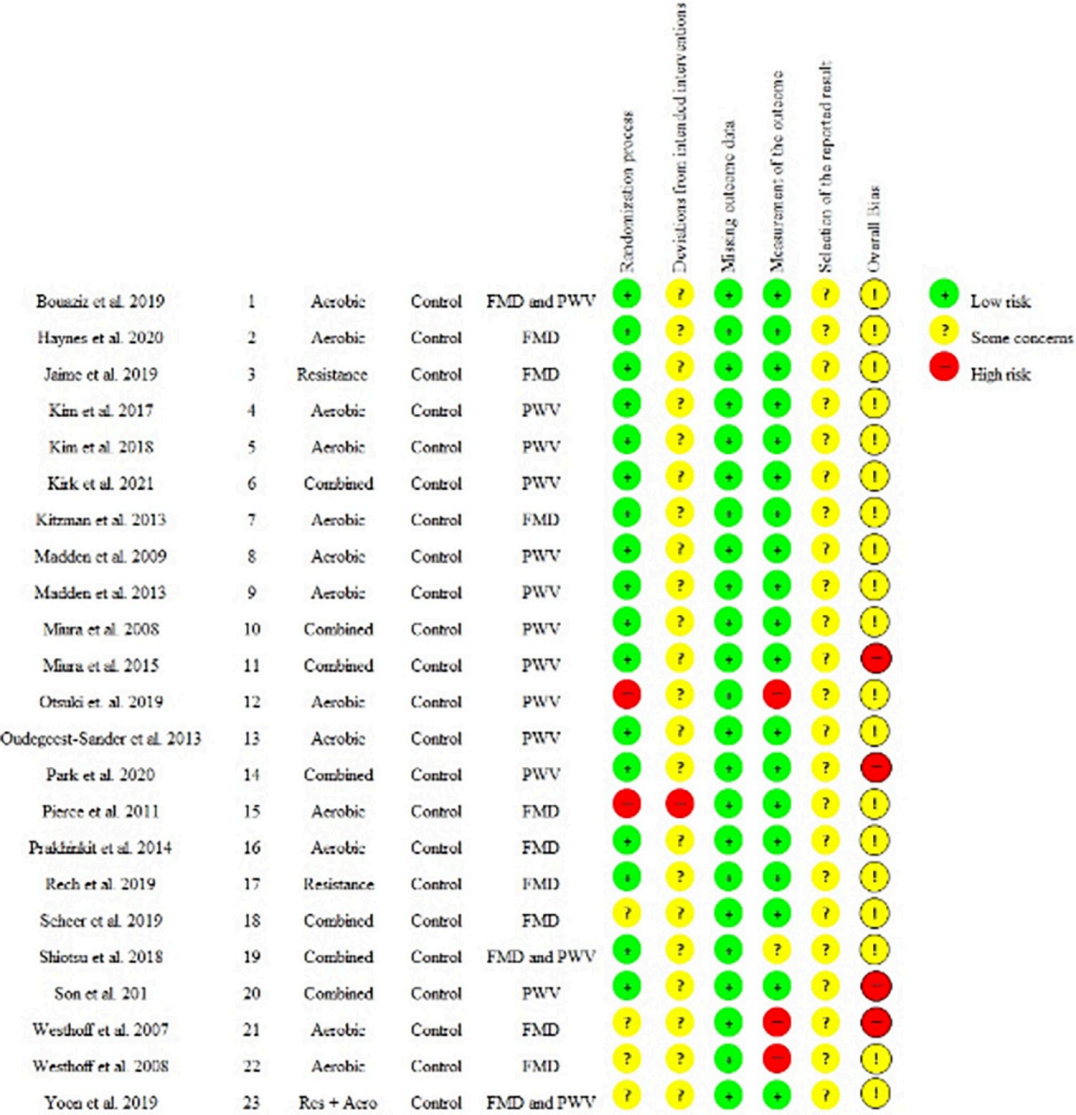
Risk of bias assessment of individual studies (using RoB2 tool).

Individual analyses of the studies showed moderate variability in the risk of publication bias (Fig 2). Twelve studies described the randomization process [26, 27, 29, 34, 38, 39, 43–46, 48, 49] and nine described blinding of evaluators to the participants’ assigned interventions [26, 30, 34, 35, 39, 45, 46, 48, 49]. One study [47] was rated as high risk of bias because the participants were assigned to groups based on their health conditions. One study [31] showed increased risk of bias because the participants were allowed to choose between the groups. And two studies [32, 33] were rated as methodologically weak as they did not report the process of randomization and blinding of evaluators. Therefore, these studies [31, 33–47] had a major impact and increased the risk of absolute bias.

Since it is difficult to blind participants to the intervention in exercise training studies, all studies were judged as some concerns of bias in the domain “deviations from intervention” and overall bias was therefore rated as “some concerns.” Yet, absolute bias was mainly judged as “some concern” because all studies analyzed did not provide detailed information in the methods to confidently answer the question, “*Were the data that produced this result analyzed in accordance with a pre-specified analysis plan that was finalized before unblinded outcome data were available for analysis*?*”* in the domain “selection of the reported result”.

A GRADE assessment of the strength of the body of evidence [50] showed moderate quality of evidence for aerobic training and resistance training (S1 and S2 Figs) and very low quality of evidence for combined training (S3 Fig) because of the very small number of studies assessing FMD included in the analysis. As for studies assessing PWV, we found moderate quality of evidence for aerobic and combined training (S1 and S3 Figs).

Although all studies included in the analysis were considered as high quality due to their design (RCT), we applied a one-point reduction in the risk of bias domain because blinding of participants to exercise training interventions is not feasible.

Moreover, we could not perform meta-regressions to examine potential effect modifiers due to the small number of studies and participants included in this analysis. Therefore, we also applied a one-point reduction in the inconsistency domain for combined and resistance training. Although subgroup analyses by health status and artery site did not show inconsistency (heterogeneity), there were few studies to support our choice of joint quality assessment of the studies (S1–S3 Figs and S1 File).

### Meta-analyses of studies assessing FMD

#### Aerobic training

**Fig 3:**
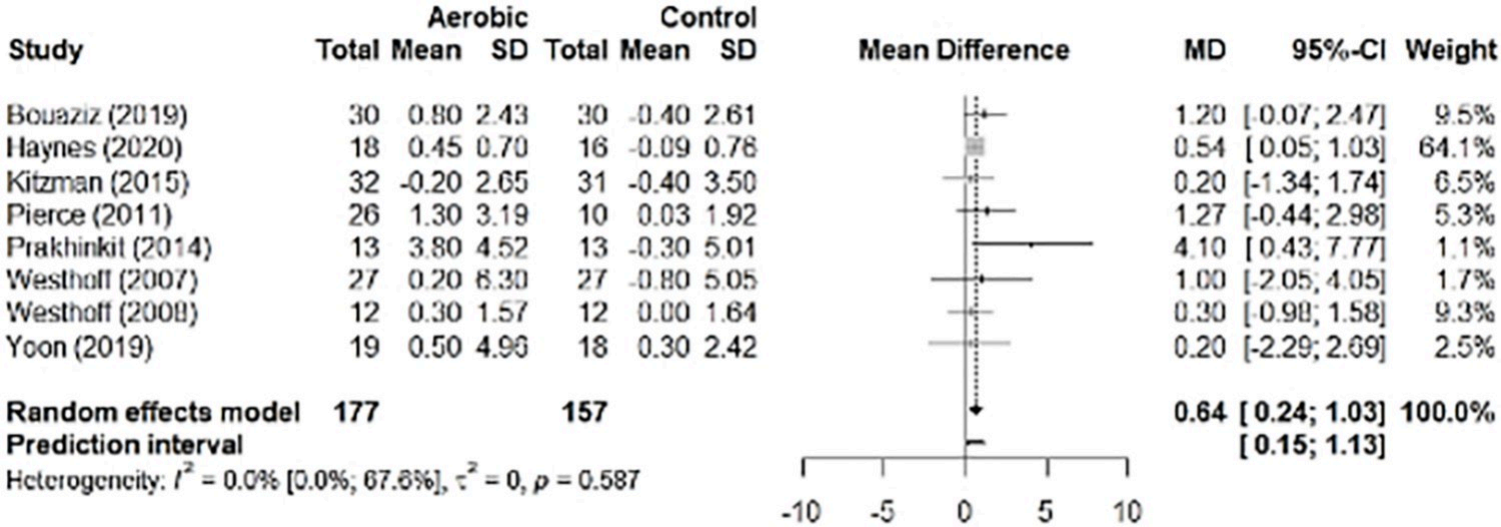
Forest plot with the effect of aerobic training (flow-mediated dilation, FMD).

The summary data of the meta-analysis of eight studies involving aerobic training (n = 325) showed absolute improvement in FMD by 0.64% (95% CI 0.24 to 1.03, p = 0.002; 95% PI 0.15 to 1.13) (Fig 3) with low heterogeneity of the studies (*I*^*2*^ 0.0% [95% CI 0.0 to 67.6%], p = 0.590). S4 Fig shows the contribution of each study to overall heterogeneity and sensitivity analysis. The subgroup analyses of the effect modifier “health condition” showed that health condition was not a determining factor for the results (healthy adults: 0.67% [95% CI 0.23 to 1.11; 95% PI –2.20 to 3.53]; adults with any health condition: 0.53% [95% CI 0.34 to 1.40; 95% PI –0.98 to 2.03]) (between groups, p = 0.777) (S5 Fig).

We were not able to perform a publication bias analysis because our study did not include the minimum number of eligible studies (n ≥ 10). In addition, given that heterogeneity was classified as “may not be important”, we did not perform a meta-regression, but conducted only subgroup analyses to explore potential heterogeneity between the studies.

#### Resistance training

**Fig 4:**
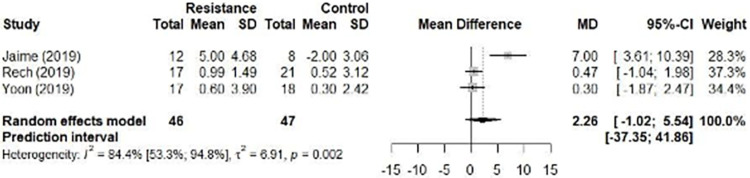
Forest plot with the effect of resistance training (flow-mediated dilation, FMD).

The meta-analysis of three studies involving resistance training (n = 93) showed no absolute improvement in FMD values (2.26% [95% CI –1.02 to 5.54], p = 0.178; 95% PI –37.35 to 41.86). The analysis of inconsistency using the Higgins test showed “considerable heterogeneity” (*I*^*2*^ 84.4% [95% CI 53.3% to 94.8], p = 0.002) (Fig 4). Given the small number of studies selected for our analysis, we were not able to perform an analysis of publication bias and meta-regression. The sensitivity analysis did not show any change in the results (S6 Fig). Yet we conducted subgroup analyses by type of resistance exercise (dynamic or isometric) and found similar results (S7 Fig).

#### Combined training

**Fig 5:**
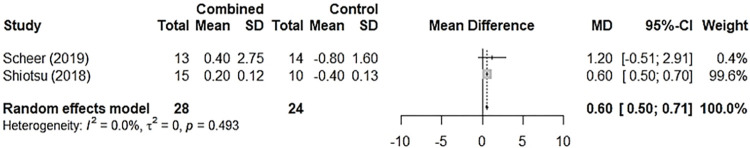
Forest plot with the effect of combined training (flow-mediated dilation, FMD).

The meta-analysis of combined training included data from only two studies (n = 52) and showed an absolute improvement in FMD by 0.60% (95% CI 0.50 to 0.71; p< 0.001). Heterogeneity was assessed as “may not be important” (*I*^*2*^ 0.0%, p = 0.493) (Fig 5). Given the small number of studies and low inconsistency, we did not perform subgroup analyses, meta-regression, publication bias or sensitivity analysis.

#### Participant adherence

In regard to adherence to the intervention protocol (analyzed vs. randomized participants), Bouaziz et al. [29] reported that 4 out of 60 participants were lost to follow-up in their study. Jaime et al. [26] reported eight lost to follow-up among 41 participants and Kitzman et al. [35] reported nine lost to follow-up among 63 participants. Pierce et al. [31] reported eight lost to follow-up among 44 participants and Prakhinkit et al. [27] reported that 5 out of the 45 participants were lost to follow-up in their study. Rech et al. [36] study identified five lost to follow-up among 44 participants and Scheer et al. [37] reported eight lost to follow-up among 35 participants. Shiotzu et al. [28] reported five lost to follow-up out of 45 participants and Westhoff et al. [32] three out of 54 participants. Yoon et al. [34] reported six lost to follow-up out of 60 participants. Westhoff et al. [33] and Haynes et al. [30] reported no participant lost to follow-up.

### Meta-analyses of studies assessing PWV

#### Aerobic training

**Fig 6:**
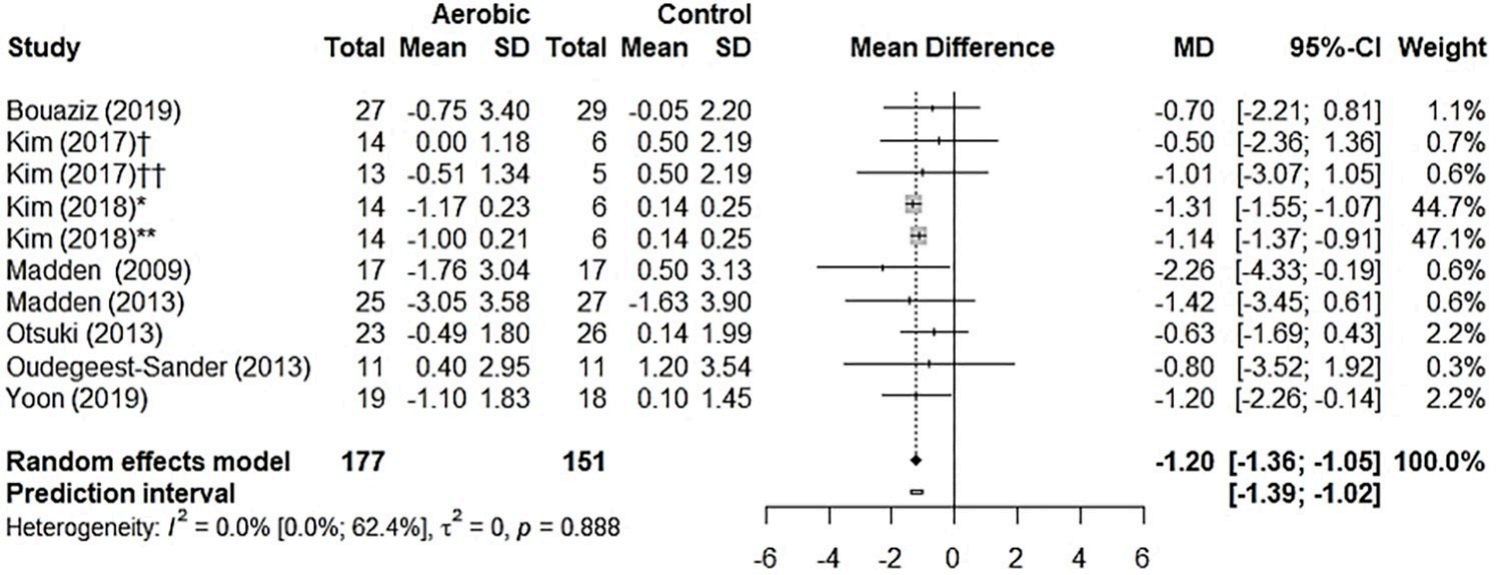
Forest plot with the effect of aerobic training (PWV).

**Fig 7:**
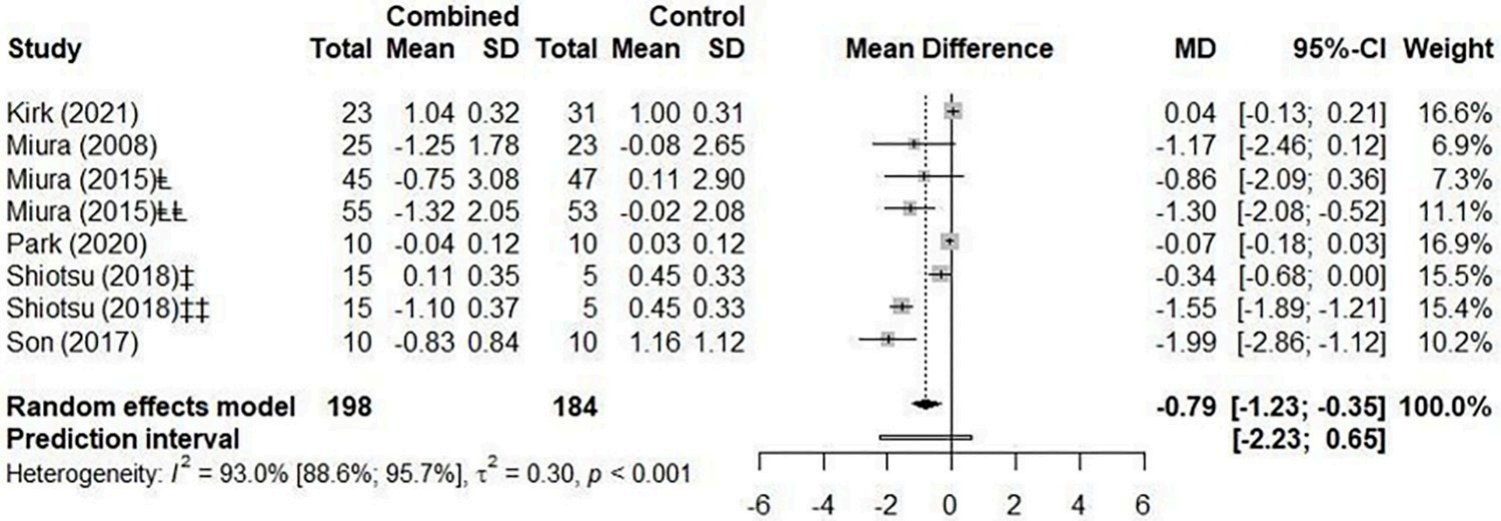
Funnel plot for risk of publication bias relative to aerobic training (pulse wave velocity, PWV).

Data from nine studies evaluating aerobic training (n = 356) showed a reduction in PWV by –1.21 m/s (95% CI –1.37 to –1.05, p< 0.001; 95% PI –1.39 to –1.03) (Fig 6). Heterogeneity was assessed as “may not be important” using the Higgins test (*I*^*2*^ 0.0% [0.0%; 62.2%], p = 0.783) (S8 Fig sensitivity analysis and contribution of each study to overall heterogeneity). The subgroup analyses by health status showed no difference between healthy adults (–0.88 m/s [95% CI –1.56 to 0.03]; 95% PI –1.84 to -0.21) and those with any condition (–1.23 m/s [95% CI –1.39 to –1.07]; 95% PI –1.39 to –1.03) (between groups, p = 0.327) (S9 Fig). The subgroup analyses by artery site showed similar reductions for the carotid-femoral artery (–1.11 m/s [95% CI –1.75 to –0.47], p< 0.001) and the carotid-brachial artery (–1.22 m/s [95% CI –1.39 to –1.06], p< 0.001). No effect was seen for the brachial-tibial artery (–1.50 m/s [95% CI –3.60 to 0.60], p = 0.081] (between groups, p = 0.913) (S10 Fig). Aerobic training for PWV did not demonstrate publication bias (p = 0.9406) (Fig 7). A meta-regression was not performed due to low inconsistency.

#### Resistance training

A pooled meta-analysis was not performed because there was not a sufficient number of studies evaluating the effect of resistance training on PWV in older adults. Only one study [34] evaluated the effect of isometric resistance training using handgrip exercise and only one study describes data for dynamic resistance training [38]. An individual analysis did not show a reduction in PWV in response to isometric resistance training (carotid-femoral artery evaluated) in the sample studied (–1.10 m/s [95% CI –2.35 to 0.15]) (S11 Fig). However, for dynamic resistance training (brachial-ankle artery evaluated) in the sample studied (–2.10 m/s [95% CI –4.12 to -0.08]) (S11 Fig).

#### Combined training

**Fig 8:**
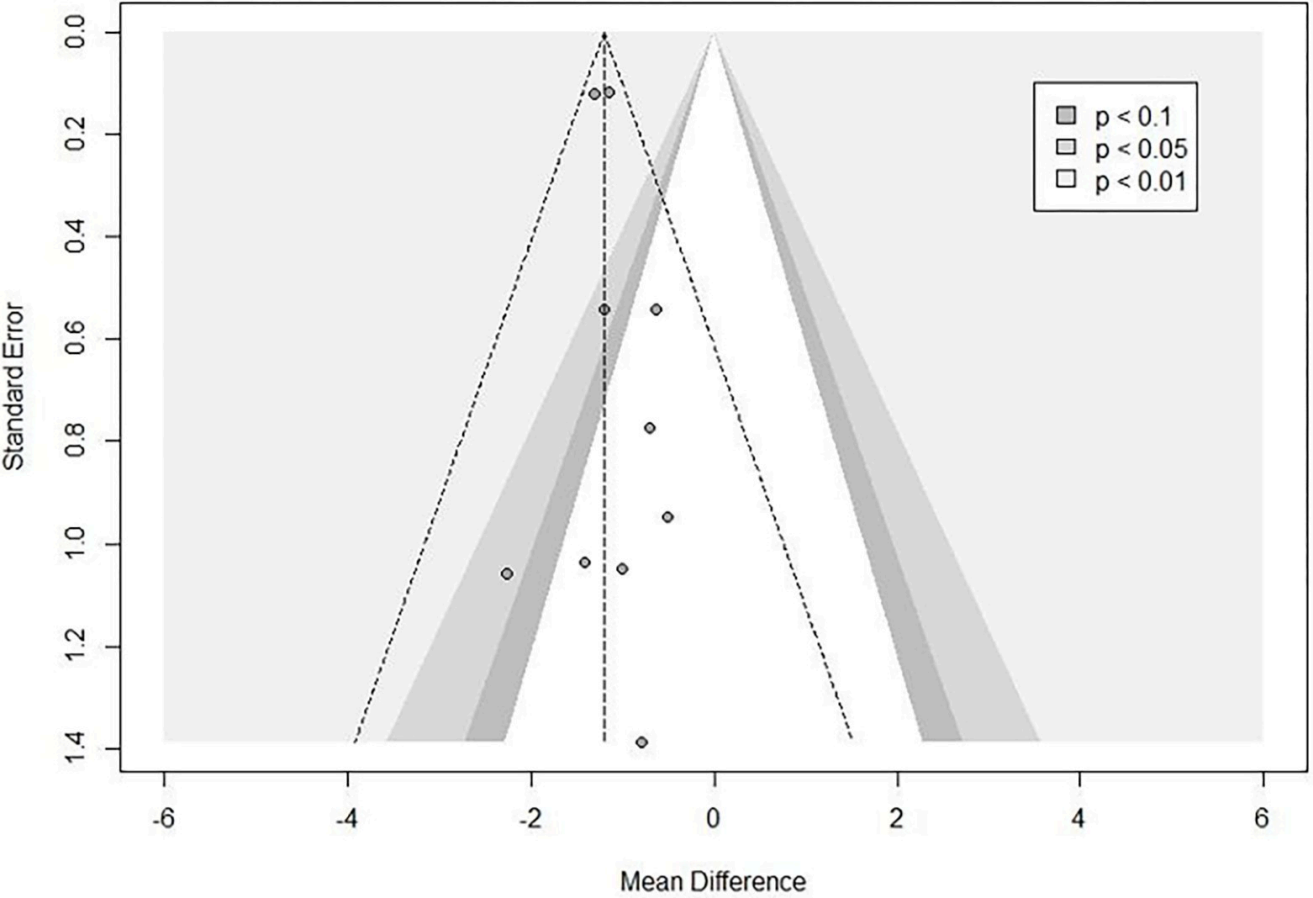
Forest plot with the effect of combined training (pulse wave velocity, PWV).

Six studies for a total of eight groups (n = 328) showed a reduction of 0.79 m/s in PWV (95% CI –1.23 to –0.35, p = 0.002; 95% PI –2.23 to 0.65 m/s) (Fig 8). The analysis of inconsistency using the Higgins test indicated “considerable heterogeneity” (*I*^*2*^ 93.0% [95% CI 88.6 to 95.7], p< 0.001). A sensitivity analysis performed where one study was removed at a time showed similar results (S12 Fig). The subgroup analyses by health status showed no difference between healthy adults and those with any condition (–0.62 m/s [95% CI –1.08 to –0.17] vs. –1.51 m/s [95% CI –2.60 to –0.42], p = 0.304) (S13 Fig). The analyses by artery site showed no differences either (brachial-tibial artery –1.04 m/s [95% CI –1.95 to –0.13]; carotid-femoral artery –0.61 m/s [95% CI –1.53 to 0.32], p = 0.902) (S14 Fig).

We performed a meta-regression analysis for potential confounders, including age, BMI, total number of days for the intervention, total amount of time in minutes for the intervention and baseline PWV values. Of the confounders evaluated, only total amount of time in minutes for the intervention (10.51%; p = 0.011) and age in the control group (73.50%; p = 0.000) were significant. The analysis can be found in the S15 Fig.

#### Participant adherence

As for adherence to the intervention (analyzed *vs*. randomized participants), Bouaziz et al. [29] reported four lost to follow-up out of 60 participants. Kim et al. [46] reported 11 out of 49. In another study, Kim et al. [39] identified eight lost to follow-up out of 48 participants. On the other hand, Kirk et al. [44] reported only one lost to follow-up out of 100. Madden et al. [45] reported only two 2 out of 36 participants. Oudegeest-Sander et al. [48] and Park et al. [43] reported four lost to follow-up out of 48. Shiotsu et al. [28] reported five lost to follow-up out of 45 and Yoon et al. [34] six out of 60. Miura et al. [41] reported 21 lost to follow-up out of 221 participants. Madden et al. [49], Son et al. [42] and Kim et al. [38] reported no participant lost to follow-up in their studies. Miura et al. [40] and Otsuki et al. [47] did not report losses.

## Discussion

The present meta-analysis involving 1,214 participants from 24 RCTs is the first to examine the effects of different training modalities on vascular function in older adults (aged ≥ 60 years). Our results showed that aerobic and combined training can improve endothelial function (assessed by FMD) and arterial stiffness (assessed by PWV) in older adults. Our results showed that aerobic and combined training can improve endothelial function (assessed by FMD), with the certainty of the evidence at "moderate" for aerobic training and "very low" for combined training in older adults. Arterial stiffness (assessed by PWV) also improves with combined training, with the evidence being "moderate" in this same population. We found improvement in FMD and PWV in response to aerobic and combined training, but this finding should be interpreted with caution as our analysis consisted of a small number of studies. In addition, the estimated PIs for aerobic training show that our results will likely to be corroborated in future RCTs.

Muscle contraction and relaxation during exercise produce a mechanical action that causes stress leading to functional adaptations within the cardiovascular system [51]. Aerobic exercise is predominantly characterized by continuous muscle contraction and relaxation in a cadenced manner for relatively long stretches of time while resistance exercise is characterized by high-intensity intermittent movements of short duration [52]. Contraction-relaxation is associated with oscillation of superficial and intramuscular blood vessel diameter with ischemia alternating with reperfusion in active and non-active muscles. Reactive hyperemia occurs along with increasing bioavailability of nitric oxide (NO) [53], which is a major contributor to FMD improvement in response to different modalities of training as seen in our study [54].

Our results point to FMD improvement following aerobic and combined training, but not in response to resistance training. This is in part corroborated by evidence reported in a meta-analysis by Ashor et al. (2015) [10] (consisting of 2,260 participants aged 18–72 years). They found a strong effect size on FMD estimated in terms of the standardized mean difference for different types of training (aerobic 2.79% [95% CI 1.2 to 3.45]; resistance 2.52% [95% CI 1.11 to 3.93]; and combined 2.07% [95% CI 0.70 to 3.44]) [10]. However, they analyzed a sample showing high heterogeneity in terms of participants’ age and health condition. In an RCT involving adults aged 54 ± 11 years with high blood pressure [55] conducted by our research team we found similar absolute improvements in FMD in response to aerobic (3.2%), resistance (4.0%) and combined training (6.8%). It is worth mentioning that an absolute difference of 1% in FMD is associated with 8–13% reduction in the risk of cardiovascular disease [56]. In another meta-analysis our group [11] evaluated the effect of aerobic training on FMD in participants aged 52–67 years with high blood pressure and found clinical improvement in FMD by 1.45%, which is of greater magnitude of effect compared to that found in this review (0.64%). A possible explanation for this finding could be the age of the studied population. In the previous study by our group (Pedralli et al.), RCTs with non-elderly individuals were also included. Unlike the present meta-analysis, which included only studies with an elderly population (≥ 60 years).

We hypothesize that the different magnitudes of FMD response to the training modalities evaluated in this study could be in part because aerobic exercise improves endothelial function by continuously increasing shear stress while resistance exercise induces intermittent increases in NO synthesis (similar to ischemia-reperfusion) [56, 57]. And that combined training consists of strength exercise plus a shorter aerobic component may explain the different magnitude of the effect of this modality on endothelial function when compared to the other two training modalities [56, 57].

In the subgroup analysis by health status, we included data from three studies involving participants with high blood pressure [32–34] and found no difference between those with any condition and healthy ones (0.53% vs. 0.67%). Some possible explanations for this finding include: albeit to a lesser extent, not everyone responds to exercise training [58]; improvement in FMD seems to be less pronounced in adults with well-established risk factors for CVDs [59]; and aging is a risk factor for vascular dysfunction and is associated with loss of endothelial integrity and increased arterial stiffness [7]. Thus, it would be logical to expect a smaller magnitude of effect in older adults with well-established risk factors.

As for the effects of resistance [26, 34, 36] and combined training [28, 37] on FMD, because the number of studies included in the analysis was small we were not able to describe more robust results and make comparisons with similar studies.

Although the small number of studies involving resistance training did not allow a more robust summary of evidence, a recent meta-analysis [60] examined the effect of resistance training on FMD in three subgroups of participants with different health status and reported a change by 2.11% in healthy participants; 2.89% in those with cardiovascular diseases; and 2.40% in those with metabolic diseases. They concluded that resistance training is an effective adjunct strategy for improving endothelial function and that individuals, regardless of their health status, can benefit from it.

The role of biochemical and physiological mechanisms has been explored and can help understand how exercise promotes direct adaptations in vascular structures and functions and explain our findings and those reported in other studies. Some of these mechanisms include increased hemodynamic forces (i.e., changes in blood pressure and transmural pressure and increased shear stress and blood flow) as a result of cyclic circumferential strain of blood vessels during muscle activity [61]; activation of endothelial mechanosensors (including membrane glycoproteins, integrins, ion channels, G*-*protein*-*coupled receptors and other specific receptors) [51]; increased expression of endothelial NO synthase (eNOS) and increased NO bioavailability associated with increased shear stress in endothelial cells [51]; reduction of free radicals (superoxide anion and peroxynitrite) and oxidizing enzymes (NADPH-oxidase and xanthine oxidase) that reduce the synthesis of eNOS and bioactivity of NO [62]; increased concentration of antioxidant enzymes, including glutathione peroxidase, superoxide dismutase and catalase [62]; reduced expression of pro-inflammatory molecules, including interleukins 8 and 10, tumor necrosis factors-alpha, cell adhesion molecules, selectins and C-reactive protein [63]; increased circulation of endothelial progenitor cells (EPCs) and increased expression of vascular endothelial growth factors (VEFG) that promote endothelial regeneration and angiogenesis [64]; and regulation of matrix metalloproteinases (MMPs) that reduces stimuli for elastin degradation and imbalances of the collagen/elastin ratio leading to arterial stiffness [65]. Regulation of MMPs is a major mechanism for preserving vascular function associated with arterial stiffness, especially during aging [65].

Our study evidenced significant clinical improvements in arterial stiffness in response to aerobic training (change by >1m/s), and a similar change following combined training. Our results corroborate the findings of two other previous meta-analyses: one by Ashor et al. (2014) [66] involving adults with mean age of 47 years and the other one by Zhang et al. (2018) [67] involving participants with well-established CVDs.

The subgroup analysis by health status showed similar PWV improvement between the groups in response to combined training. On the other hand, meta-analyses involving healthy individuals [14], individuals with arterial hypertension [19] and other types of CVDs [67] reported no effect of resistance training on PWV.

A major limitation of this study is that the analysis included only one study [34] evaluating the effect of resistance training on PWV in older adults and no inferences could be made from the data. However, an individual analysis showed a positive effect of handgrip (isometric) resistance exercise in participants with systemic arterial hypertension, which is in line with that reported by Lopes et al. (2021) [19]. These authors found in their study a reduction of PWV in individuals with hypertension in response to isometric resistance training. Another point worth mentioning is that we were not able to perform additional analyses such as correlation analyses between FMD and PWV because our review included only three studies involving both outcomes but they used different training modalities [25, 26, 31]. In addition, we did not perform a subgroup analysis of vascular bed assessed for PWV by resistance training as there were only two studies included with different modalities—dynamic and isometric exercise.

Carotid-femoral PWV is the reference measure of arterial stiffness. It is assessed in large central arteries and is more directly associated with left ventricle overload (high augmentation index, pulse pressure and reflected wave velocity) as well with overload pressure in the coronary arteries and arteries supplying blood to the brain, and can more accurately predict the risk of target organ damage and associated event [68]. However, studies have shown strong predictive correlation with different artery sites evaluated [69] demonstrating similar predictive values for CV morbidity and mortality and deaths from all causes [9]. Our meta-analysis found similar effects on carotid-femoral and carotid-brachial PWV in response to aerobic training. Combined training promoted similar effects on carotid-femoral and carotid-brachial PWV. Given that a reduction by –1 m/s is related to a relative risk reduction of up to 15% in deaths from CVDs [9], we can infer that our results indicate major benefits of aerobic training and potential clinical benefits of combined training associated with reduced risk of fatal cardiovascular events.

Overall, we included in our analysis 15 studies evaluating the effect of aerobic training, six studies evaluating the effect of combined training and only four evaluating the effect of resistance training. The relatively small number of studies for each training modality makes it difficult to compare our results with descriptive findings of previous studies. Moreover, these studies evaluated different populations that were heterogeneous in terms of age and/or health status. Few studies in the literature have evaluated the effect of resistance and combined training on FMD and PWV in older adults and more research is needed to further examine these outcomes. Hence, any comparison and extrapolation of our results must be done with extreme caution based on physiological and methodological considerations as well as taking into account variables such as age, sample size and health conditions.

Lastly, FITT data extracted from the studies selected for the analysis can be used to guide the prescription of aerobic training for older adults. For improving FMD, an exercise frequency of at least three times a week at intensity of 55% HRR or 70% HRmax for 40 minutes is recommended. Likewise, aerobic exercise at an intensity corresponding to 60% HRR or 70% HRmax for 50 minutes at a frequency of three times a week or more is recommended to reduce arterial stiffness. In addition, based on our results and known functional benefits of resistance training, combined training is another approach for prescribing exercise to improve cardiovascular health in older adults.

## Conclusions

Our systematic review of RCTs showed that aerobic and combined training can promote major improvements in endothelial function and arterial stiffness, measured by FMD and PWV, in older adults. Healthy adults do not seem to benefit more from aerobic and combined training compared to those with comorbidities. Our findings for combined and resistance training should be interpreted with caution as very few studies examined the effects of these training modalities on FMD and PWV in older adults. We recommend more research to further understand the benefits of exercise interventions.

## Supporting information

S1 Checklist PRISMA 2020 checklist. (DOCX)

S2 Checklist PRISMA 2020 for abstracts checklist. (DOCX)

S1 File Containing S1 and S2 Charts. (DOCX)

S1 Fig Aerobic training table. (TIFF)

S2 Fig Resistance training table. (TIFF)

S3 Fig Combined training table. (TIFF)

S4 Fig (TIFF)

S5 Fig Forest plot with the effect of aerobic training in subgroups by health status (flow-mediated dilation, FMD). (TIFF)

S6 Fig Forest plot with the effect of resistance training on flow-mediated dilation (FMD) in subgroups by exercise type (resistance or isometric). (TIFF)

S7 Fig Contribution to overall heterogeneity of studies involving combining training and flow-media ted dilatation (FMD) in the meta-analysis. (TIFF)

S8 Fig Forest plot with the effect of aerobic training on pulse wave velocity (PWV) in subgroups by health status. (TIFF)

S9 Fig Forest plot with the effect of aerobic training on pulse wave velocity (PWV) in subgroups by artery site. (TIFF)

S10 Fig Individual effect of resistance training on pulse wave velocity in Yoon study (2019). (TIFF)

S11 Fig Sensitivity analysis for the FMD variable in resistance training. (TIFF)

S12 Fig Forest plot with the effect of combined training on pulse wave velocity (PWV) in subgroups by health status. (TIFF)

S13 Fig Forest plot with the effect of combined training on pulse wave velocity (PWV) in subgroups by artery site. (TIFF)

S14 Fig Meta regression of possible confounding factors. BMI: body mass index.
(TIFF)

S15 Fig Meta-regression analysis for potential confounders, including age, BMI, total number of days for the intervention, total amount of time in minutes for the intervention and baseline PWV values for combined training. (JPEG)
