# Supplementary material for: Effects of aerobic, resistance and combined training on endothelial function and arterial stiffness in older adults: A systematic review and meta-analysis
Source: PLoS One. 2024 Dec 2;19(12):e0308600. doi: 10.1371/journal.pone.0308600 (PMC11611152; doi:10.1371/journal.pone.0308600)
Supplement: S1 File — (DOCX) [file pone.0308600.s003.docx]

**Supplementary Material**

**EFFECTS OF AEROBIC, RESISTANCE AND COMBINED TRAINING ON ENDOTHELIAL FUNCTION AND ARTERIAL STIFFNESS IN OLDER ADULTS: A SYSTEMATIC REVIEW AND META-ANALYSIS**

Raphael S. N da Silva^1^, Diego S. da Silva^1^, Patrícia C. de Oliveira^1^,

Gustavo Waclawovsky^1^ & Maximiliano I. Schaun^1^

^1^ Instituto de Cardiologia do Rio Grande do Sul/Fundação Universitária de Cardiologia, Porto Alegre, Brasil.

**Corresponding author:**

Dr. Maximiliano I Schaun

Instituto de Cardiologia do Rio Grande do Sul/Fundação Universitária de Cardiologia

Av. Princesa Isabel, 395 Santana, 90620-001 Porto Alegre – RS Brazil

Phone:+55(51)32303600, branch 3636/3757

E-mail: [dr.maxschaun@gmail.com](mailto:dr.maxschaun@gmail.com)

**Chart 1S.** Search strategies for each database

| **Medline (PubMed)** |
| --- |
| ***((EXERCISE [MeSH Terms]*** *OR Exercises OR Physical Activity OR Activities, Physical OR Activity, Physical OR Physical Activities OR Exercise, Physical OR Exercises, Physical OR Physical Exercise OR Physical Exercises OR Acute Exercise OR Acute Exercises OR Exercise, Acute OR Exercises, Acute OR Exercise, Isometric OR Exercises, Isometric OR Isometric Exercises OR Isometric Exercise OR Exercise, Aerobic OR Aerobic Exercise OR Aerobic Exercises OR Exercises, Aerobic OR Exercise Training OR Exercise Trainings OR Training, Exercise OR Trainings, Exercise)* ***OR (Resistance Training [MeSH Terms]*** *OR Training, Resistance OR Strength Training OR Training, Strength OR Weight-Lifting Strengthening Program OR Strengthening Program, Weight-Lifting OR Strengthening Programs, Weight-Lifting OR Weight Lifting Strengthening Program OR Weight-Lifting Strengthening Programs OR Weight-Lifting Exercise Program OR Exercise Program, Weight-Lifting OR Exercise Programs, Weight-Lifting OR Weight Lifting Exercise Program OR Weight-Lifting Exercise Programs OR Weight-Bearing Strengthening Program OR Strengthening Program, Weight-Bearing OR Strengthening Programs, Weight-Bearing OR Weight Bearing Strengthening Program OR Weight-Bearing Strengthening Programs OR Weight-Bearing Exercise Program OR Exercise Program, Weight-Bearing OR Exercise Programs, Weight-Bearing OR Weight Bearing Exercise Program OR Weight-Bearing Exercise Programs)* ***OR*** ***(Endurance Training [MeSH Terms]*** *OR Training, Endurance****))*** ***AND ((Vascular Endothelium*** ***[MeSH Terms]*** *OR Endothelium, Vascular* *OR Endotheliums, Vascular OR Vascular Endotheliums OR Capillary Endothelium OR Capillary Endotheliums OR Endothelium, Capillary OR Endotheliums, Capillary)* ***OR (Vasodilation [MeSH Terms]*** *OR Vasorelaxation OR Vasodilatation OR Vascular Endothelium-Dependent Relaxation OR Endothelium-Dependent Relaxation, Vascular OR Relaxation, Vascular Endothelium-Dependent OR Vascular Endothelium Dependent Relaxation)* ***OR (Hyperemia [MeSH Terms] OR*** *Hyperemias OR Active Hyperemia OR Hyperemia, Active OR Arterial Hyperemia OR Hyperemia, Arterial OR Venous Engorgement OR Engorgement, Venous OR Venous Congestion OR Congestion, Venous OR Passive Hyperemia OR Hyperemia, Passive OR Reactive Hyperemia OR Hyperemia, Reactive OR Hyperemias, Reactive OR Reactive Hyperemias)* ***AND (Vascular Stiffness [MeSH Terms] OR*** *Vascular OR Vascular Stiffnesses OR Arterial Stiffness OR Arterial Stiffnesses OR Stiffness, Arterial OR Aortic Stiffness OR Aortic Stiffnesses OR Stiffness, Aortic)* ***OR*** ***(Pulse Wave Analysis [MeSH Terms]*** *OR Analyses, Pulse Wave OR Analysis, Pulse Wave OR Pulse Wave Analyses OR Wave Analyses, Pulse OR Wave Analysis, Pulse OR Pulse Wave Velocity OR Pulse Wave Velocities OR Velocities, Pulse Wave OR Velocity, Pulse Wave OR Wave Velocities, Pulse OR Wave Velocity, Pulse OR Pulse Transit Time OR Pulse Transit Times OR Time, Pulse Transit OR Times, Pulse Transit OR Transit Time, Pulse OR Transit Times, Pulse OR Pulse Wave Transit Time****))*** ***AND (RANDOMIZED CONTROLLED TRIAL[pt]*** *OR controlled clinical trial[pt] OR randomized controlled trials[mh] OR random allocation[mh] OR double-blind method[mh] OR single-blind method[mh] OR clinical trial[pt] OR clinical trials[mh] OR “clinical trial”[tw] OR singl*[tw] OR doubl*[tw] OR trebl*[tw] OR tripl*[tw] OR random*[tw] OR cross-over studies[mh] OR control*[tw] OR volunteer*[tw])* |
| **COCHRANE** |
| *((Exercise) OR (Physical Activity) OR (Training)) AND ((Endothelium) OR (Vasodilation) OR (Hyperemia)) AND ((Vascular Stiffness) OR (Arterial Stiffness) OR (Pulse Wave Analysis))* |
| **Web of Science** |
| *TS=(exercise OR training OR physical activity) AND TS=(endothelium OR vasodilation OR vasorelaxation OR hyperemia) AND TS=(vascular OR arterial OR pulse wave velocity OR stitffness) AND TS=(randomized controlled trial OR randomized controlled trial)* |
| **EMBASE** |
| *('exercise'/exp OR 'exercise' OR 'training'/exp OR 'training' OR 'physical activity'/exp OR 'physical activity') AND ('vascular endothelium'/exp OR 'vascular endothelium' OR 'hyperemia'/exp OR 'hyperemia' OR 'vasodilatation'/exp OR 'vasodilatation') AND (vascular OR 'arterial stiffness'/exp OR 'arterial stiffness' OR 'pulse wave'/exp OR 'pulse wave') AND ('randomized controlled trial'/exp OR 'randomized controlled trial')* |
| **LILACS (Portuguese)** |
| (tw:(**Exercício Físico**)) AND (tw**:(Endotélio**)) OR (tw:(**Endotélio Vascular**)) OR (tw:(**Vasodilatação**)) OR (tw:(**hiperemia**)) AND (tw:(**Rigidez vascular**)) OR (tw:(**Análise de Onda de Pulso**)) |
| **LILACS (Spanish)** |
| (tw:(**Ejercicio físico**)) AND (tw**:(Endotelio**)) OR (tw:(**Endotelio Vascular**)) OR (tw:(**Vasodilatación**)) OR (tw:(**hiperemia**)) AND (tw:(**Rigidez vascular**)) OR (tw:(**Análisis de la onde del pulso**)) |
| **LILACS (English)** |
| (tw:(**Exercise**)) AND (tw**:(Endothelium**)) OR (tw:(**Endothelium, Vascular**)) OR (tw:(**Vasodilation**)) OR (tw:(**hyperemia**)) AND (tw:(**Vascular Stiffness**)) OR (tw:(**Pulse Wave Analysis**)) |
| **OpenGray** |
| *(exercise OR training OR resistance training OR endurance training) AND (endothelium OR vascular endothelium OR vascular stiffness OR arterial stiffness)* |
| **CAPES thesis bank** |
| *(exercício físico OR treinamento físico) AND (função vascular)* |
| **Brazilian Clinical Trials Registry** |
| (exercício físico OR treinamento físico) AND (função vascular) |
| **ClinicalTrials.gov** |
| **Search 01*:*** *endothelial function AND exercise;* **search 02:** *endotelial function and training zero*; **search 03:** *arterial stiffness and exercise*; **search 04:** *arterial stiffness and training* |
| **WHO** |
| **Search 01*:*** *endothelial function AND exercise;* **search 02:** *endotelial function and training zero*; **search 03:** *arterial stiffness and exercise*; **search 04:** *arterial stiffness and training* |

**Chart 2S.** Script for the meta-analysis of studies assessing FMD

| **# FMD aerobic training**   - Library (readxl) - FMD_aerobic <- read_excel ("Bancos de dados/FMD_aerobic.xlsx") - View (FMD_aerobic) - meta_Rapha1 = FMD_aerobic <- metacont (tt_n, tt_mean, tt_dp, c_n, c_mean, c_dp, Study, predict =TRUE, data = FMD_aerobic, sm = "MD") - meta_Rapha1 = FMD_aerobic <- metacont (tt_n, tt_mean, tt_dp, c_n, c_mean, c_dp, Study, predict =TRUE, byvar = Condition, data = FMD_aerobic, sm = "MD") - meta_Rapha1 - forest (meta_Rapha1, sortvar = Study, xlim = c (-7.0, 5.0), predict = TRUE, col.square = "grey", col.diamond = "black", digits = 2) - forest (meta_Rapha1, comb.fixed = FALSE, sortvar = Study, xlim = c (-12.0, 12.0), digits.sd = 2, digits.I2 = 1, print.I2.ci = TRUE , digits.tau2 = 2, digits.pval.Q = 3, squaresize = 0.5, lab.e = "Aerobic", lab.c = "Control", col.inside = "black", col.square = "grey", col.diamond = "black", col.predict = "transparent", digits = 2) - baujat (meta_Rapha1, ylim = c (-0.65, 0.65), xlim = c (-200, 200)) - metainf (meta_Rapha1, pooled = "random") - metabias (meta_Rapha1, method.bias = "linreg", k.min= 8) - funnel (meta_Rapha1) - col.contour = c ("gray75", "gray85", "gray95") - funnel.meta (meta_Rapha1, xlim = c (-12.0, 12.0), contour = c (0.9, 0.95, 0.99), col.contour = col.contour) - legend (x = 9.5, y = 0.10, legend = c ("p < 0.1", "p < 0.05", "p < 0.01"), fill = col.contour)   **# FMD combined training**   - library (readxl) - FMD_combined <- read_excel ("Bancos de dados/FMD_combined.xlsx") - View (FMD_combined) - meta_Rapha2 = FMD_combined <- metacont (tt_n, tt_mean, tt_dp, c_n, c_mean, c_dp, Study, predict =TRUE, data = FMD_combined, sm = "MD") - meta_Rapha2 - forest (meta_Rapha2, sortvar = Study, xlim = c (-7.0, 5.0), predict = TRUE, col.square = "grey", col.diamond = "black", digits = 2) - forest (meta_Rapha2, comb.fixed = FALSE, sortvar = Study, xlim = c (-12.0, 12.0), digits.sd = 2, digits.I2 = 1, print.I2.ci = TRUE , digits.tau2 = 2, digits.pval.Q = 3, squaresize = 0.5, lab.e = "Combined", lab.c = "Control", col.inside = "black", col.square = "grey", col.diamond = "black", col.predict = "transparent", digits = 2) - baujat (meta_Rapha2, ylim = c (-0.65, 0.65), xlim = c (-200, 200)) - metainf (meta_Rapha2, pooled = "random")   **# FMD resistance training**   - library (readxl) - FMD_resistance <- read_excel ("Bancos de dados/FMD_resistance.xlsx") - View (FMD_resistance) - meta_Rapha3 = FMD_resistance <- metacont (tt_n, tt_mean, tt_dp, c_n, c_mean, c_dp, Study, predict =TRUE, data = FMD_resistance, sm = "MD") - meta_Rapha3 = FMD_resistance <- metacont (tt_n, tt_mean, tt_dp, c_n, c_mean, c_dp, Study, byvar = Intervention, predict =TRUE, data = FMD_resistance, sm = "MD") - meta_Rapha3 - forest (meta_Rapha3, sortvar = Study, xlim = c (-7.0, 5.0), predict = TRUE, col.square = "grey", col.diamond = "black", digits = 2) - forest (meta_Rapha3, comb.fixed = FALSE, sortvar = Study, xlim = c (-18.0, 18.0), digits.sd = 2, digits.I2 = 1, print.I2.ci = TRUE , digits.tau2 = 2, digits.pval.Q = 3, squaresize = 0.5, lab.e = "Resistance", lab.c = "Control", col.inside = "black", col.square = "grey", col.diamond = "black", col.predict = "transparent", digits = 2) - baujat (meta_Rapha3, ylim = c (-0.65, 0.65), xlim = c (-200, 200)) - metainf (meta_Rapha3, pooled = "random")   **# PWV aerobic training**   - library (readxl) - PWV_aerobic <- read_excel("Bancos de dados/PWV_aerobic.xlsx") - View (PWV_aerobic) - meta_Rapha4 = PWV_aerobic <- metacont (tt_n, tt_mean, tt_dp, c_n, c_mean, c_dp, Study, predict =TRUE, data = PWV_aerobic, sm = "MD") - meta_Rapha4 = PWV_aerobic <- metacont (tt_n, tt_mean, tt_dp, c_n, c_mean, c_dp, Study, predict =TRUE, byvar = Condition, data = PWV_aerobic, sm = "MD") - meta_Rapha4 = PWV_aerobic <- metacont (tt_n, tt_mean, tt_dp, c_n, c_mean, c_dp, Study, predict =TRUE, byvar = Location, data = PWV_aerobic, sm = "MD") - meta_Rapha4 - forest (meta_Rapha4, sortvar = Study, xlim = c (-6.0, 6.0), predict = TRUE, col.square = "grey", col.diamond = "black", digits = 2) - forest (meta_Rapha4, comb.fixed = FALSE, sortvar = Study, xlim = c (-6.0, 6.0), digits.sd = 2, digits.I2 = 1, print.I2.ci = TRUE , digits.tau2 = 2, digits.pval.Q = 3, squaresize = 0.5, lab.e = "Aerobic", lab.c = "Control", col.inside = "black", col.square = "grey", col.diamond = "black", col.predict = "transparent", digits = 2) - baujat (meta_Rapha4, ylim = c (-0.65, 0.65), xlim = c (-200, 200)) - metainf (meta_Rapha4, pooled = "random") - metabias (meta_Rapha4, method.bias = "linreg", k.min= 8) - funnel (meta_Rapha4) - col.contour = c ("gray75", "gray85", "gray95") - funnel.meta (meta_Rapha4, xlim = c(-6.0, 6.0), contour = c(0.9, 0.95, 0.99), col.contour = col.contour) - legend (x = 3.5, y = 0.10, legend = c ("p < 0.1", "p < 0.05", "p < 0.01"), fill = col.contour)   **# PWV combined training**   - library (readxl) - PWV_combined <- read_excel ("Bancos de dados/PWV_combined.xlsx") - View (PWV_combined) - meta_Rapha6 = PWV_combined <- metacont (tt_n, tt_mean, tt_dp, c_n, c_mean, c_dp, Study, predict =TRUE, data = PWV_combined, sm = "MD") - meta_Rapha6 <- metacont(tt_n,tt_mean,tt_dp,c_n,c_mean,c_dp, Study, predict=TRUE, byvar = Condition, data = PWV_combined , sm="MD") - meta_Rapha6 <- metacont(tt_n,tt_mean,tt_dp,c_n,c_mean,c_dp, Study, predict=TRUE, byvar = Location, data = PWV_combined , sm="MD") - meta_Rapha6 - forest (meta_Rapha6, sortvar = Study, xlim = c (-7.0, 5.0), predict = TRUE, col.square = "grey", col.diamond = "black", digits = 2) - forest (meta_Rapha6, comb.fixed = FALSE, sortvar = Study, xlim = c (-6.0, 6.0), digits.sd = 2, digits.I2 = 1, print.I2.ci = TRUE , digits.tau2 = 2, digits.pval.Q = 3, squaresize = 0.5, lab.e = "Combined", lab.c = "Control", col.inside = "black", col.square = "grey", col.diamond = "black", col.predict = "transparent", digits = 2) - baujat (meta_Rapha6, ylim = c (-0.65, 0.65), xlim = c (-200, 200)) - metainf (meta_Rapha6, pooled = "random") - metabias (meta_Rapha6, method.bias = "linreg", k.min= 8) - funnel (meta_Rapha6) - col.contour = c ("gray75", "gray85", "gray95") - funnel.meta (meta_Rapha6, xlim = c (-15.0, 15.0), contour = c (0.9, 0.95, 0.99), col.contour = col.contour) - legend (x = 9.5, y = 0.10, legend = c ("p < 0.1", "p < 0.05", "p < 0.01"), fill = col.contour) - meta_Rapha7 = subset (PWV_combined, Study!= "Miura (2008)") - meta_Rapha7 = subset (PWV_combined, Study!= "Miura (2015)Ⱡ") - meta_Rapha7 = subset (PWV_combined, Study!= "Miura (2015)ⱠⱠ") - meta_Rapha7 = subset (PWV_combined, Study!= "Park (2020)") - meta_Rapha7 = subset (PWV_combined, Study!= "Son (2017)") - meta_Rapha7 = subset (PWV_combined, Study!= "Shiotsu (2018)‡") - meta_Rapha7 = subset (PWV_combined, Study!= "Shiotsu (2018)‡‡") - View (meta_Rapha7) - meta_Rapha7 <- metacont (tt_n,tt_mean,tt_dp,c_n,c_mean,c_dp, Study, predict=TRUE, data = meta_Rapha7 , sm="MD") - meta_Rapha7 <- metacont (tt_n,tt_mean,tt_dp,c_n,c_mean,c_dp, Study, predict=TRUE, byvar = Condition, data = meta_Rapha7 , sm="MD") - meta_Rapha7 - forest (meta_Rapha7, sortvar = Study, xlim = c (-7.0, 5.0), predict = TRUE, col.square = "grey", col.diamond = "black", digits = 2) - forest (meta_Rapha7, comb.fixed = FALSE, sortvar = Study, xlim = c (-6.0, 6.0), digits.sd = 2, digits.I2 = 1, print.I2.ci = TRUE , digits.tau2 = 2, digits.pval.Q = 3, squaresize = 0.5, lab.e = "Combined", lab.c = "Control", col.inside = "black", col.square = "grey", col.diamond = "black", col.predict = "transparent", digits = 2) - total_frequency<- (c (48.0,24.0,24.0,24.0,36.0,20.0,20.0,36.0)) - qqnorm (total_frequency) - qqPlot (total_frequency) - qqline (total_frequency,col = "Black", lwd = 2) - shapiro.test (total_frequency) - plot (total_frequency, residuals(meta_Rapha6)) - abline (h=0) - metareg (meta_Rapha6, ~total_frequency) - meta_Rapha6 <- metareg (meta_Rapha6, ~total_frequency) - bubble (meta_Rapha6, col.line = "blue",col = "black", studlab = TRUE) - total_time_minutes<- (c (3456,960,960,960,3240,800,800,2520)) - qqnorm (total_time_minutes) - qqPlot (total_time_minutes) - qqline (total_time_minutes,col = "Black", lwd = 2) - shapiro.test (total_time_minutes) - plot (total_time_minutes, residuals(meta_Rapha6)) - abline (h=0) - metareg (meta_Rapha6, ~total_time_minutes) - meta_Rapha6 <- metareg (meta_Rapha6, ~total_time_minutes) - bubble (meta_Rapha6, col.line = "blue",col = "black", studlab = TRUE) - age_t_mean <- (c (66.63, 69.50, 72.90, 72.0, 69.10, 70.40, 69.60, 76.50)) - qqnorm (age_t_mean, pch = 1) - qqline (age_t_mean,col = "Black", lwd = 2) - qqPlot (age_t_mean) - shapiro.test (age_t_mean) - plot (age_t_mean, residuals (meta_Rapha6)) - abline (h=0) - metareg (meta_Rapha6, ~age_t_mean) - meta_Rapha6 <- metareg (meta_Rapha6, ~age_t_mean) - bubble (meta_Rapha6, col.line = "blue",col = "black", studlab = TRUE) - age_c_mean <- (c (68.16, 68.90, 69.70, 71.80, 68.50, 71.0,71.0, 74.70)) - qqnorm (age_c_mean, pch = 1) - qqline (age_c_mean,col = "Black", lwd = 2) - qqPlot (age_c_mean) - shapiro.test (age_c_mean) - plot (age_c_mean, residuals (meta_Rapha6)) - abline (h=0) - metareg (meta_Rapha6, ~age_c_mean) - meta_Rapha6 <- metareg (meta_Rapha6, ~age_c_mean) - bubble (meta_Rapha6, col.line = "blue",col = "black", studlab = TRUE) - BMI_t <- (c (22.80, 22.47, 22.47, 26.20, 23.60, 24.10, 24.71)) - qqnorm (BMI_t, pch = 1) - qqline (BMI_t,col = "Black", lwd = 2) - qqPlot (BMI_t) - shapiro.test (BMI_t) - plot (BMI_t, residuals (BMI_t)) - abline (h=0) - metareg (meta_Rapha6, ~BMI_t) - BMI_t <- metareg (meta_Rapha6, ~BMI_t) - bubble (BMI_t, col.line = "blue",col = "black", studlab = TRUE) - BMI_c <- (c (23.70, 23.29, 23.29, 26.00, 23.80, 23.80, 24.05)) - qqnorm (BMI_c, pch = 1) - qqline (BMI_c,col = "Black", lwd = 2) - qqPlot (BMI_c) - shapiro.test (BMI_c) - plot (BMI_c, residuals (BMI_c)) - abline (h=0) - metareg (meta_Rapha6, ~BMI_c) - BMI_c <- metareg (meta_Rapha6, ~BMI_c) - bubble (BMI_c, col.line = "blue",col = "black", studlab = TRUE) - tt_mean_BEFORE <- (c (18.96, 17.08, 16.84, 15.98, 9.45, 8.78, 8.34)) - qqnorm (tt_mean_BEFORE, pch = 1) - qqline (tt_mean_BEFORE,col = "Black", lwd = 2) - qqPlot (tt_mean_BEFORE) - shapiro.test (tt_mean_BEFORE) - plot (tt_mean_BEFORE, residuals (tt_mean_BEFORE)) - abline (h=0) - metareg (meta_Rapha6, ~tt_mean_BEFORE) - tt_mean_BEFORE <- metareg (meta_Rapha6, ~tt_mean_BEFORE) - bubble (tt_mean_BEFORE, col.line = "blue",col = "black", studlab = TRUE) - c_mean_BEFORE <- (c (18.31, 17.04, 16.43, 15.73, 8.60, 8.00, 8.00)) - qqnorm (c_mean_BEFORE, pch = 1) - qqline (c_mean_BEFORE,col = "Black", lwd = 2) - qqPlot (c_mean_BEFORE) - shapiro.test (c_mean_BEFORE) - plot (c_mean_BEFORE, residuals (c_mean_BEFORE)) - abline (h=0) - metareg (meta_Rapha6, ~c_mean_BEFORE) - c_mean_BEFORE <- metareg (meta_Rapha6, ~c_mean_BEFORE) - bubble (c_mean_BEFORE, col.line = "blue",col = "black", studlab = TRUE)   **# PWV resistance training**   - library (readxl) - PWV_resistance <- read_excel ("Bancos de dados/PWV_resistance.xlsx") - View (PWV_resistance) - meta_Rapha8 = PWV_resistance <- metacont (tt_n, tt_mean, tt_dp, c_n, c_mean, c_dp, Study, predict =TRUE, data = PWV_resistance, sm = "MD")   meta_Rapha8 |
| --- |

**Chart 15S. Meta-regression analysis for potential confounders, including age, BMI, total number of days for the intervention, total amount of time in minutes for the intervention and baseline PWV values for combined training.**

| ***Moderators*** | ***Study (n)*** | ***p-value*** | ***Heterogeneity accounted (R^2^)*** |
| --- | --- | --- | --- |
| **Age (group intervetion)** | **8** | **0.0152** | **------** |
| **Age (group control)** | **8** | **< 0.0001** | **73.50%** |
| **BMI (group intervation)** | **7** | **0.2805** | **------** |
| **BMI (group control)** | **7** | **0.2860** | **------** |
| **Total frequency (days)** | **8** | **0.0701** | **------** |
| **Total time (minutes)** | **8** | **0.0111** | **10.51%** |
| **Pulse wave velocity (group intervention)** | **8** | **0.6417** | **------** |
| **Pulse wave velocity (group control)** | **8** | **0.7077** | **------** |
