# Supplementary figures and images for: Effects of aerobic, resistance and combined training on endothelial function and arterial stiffness in older adults: A systematic review and meta-analysis
Source: PLoS One. 2024 Dec 2;19(12):e0308600. doi: 10.1371/journal.pone.0308600 (PMC11611152; doi:10.1371/journal.pone.0308600)

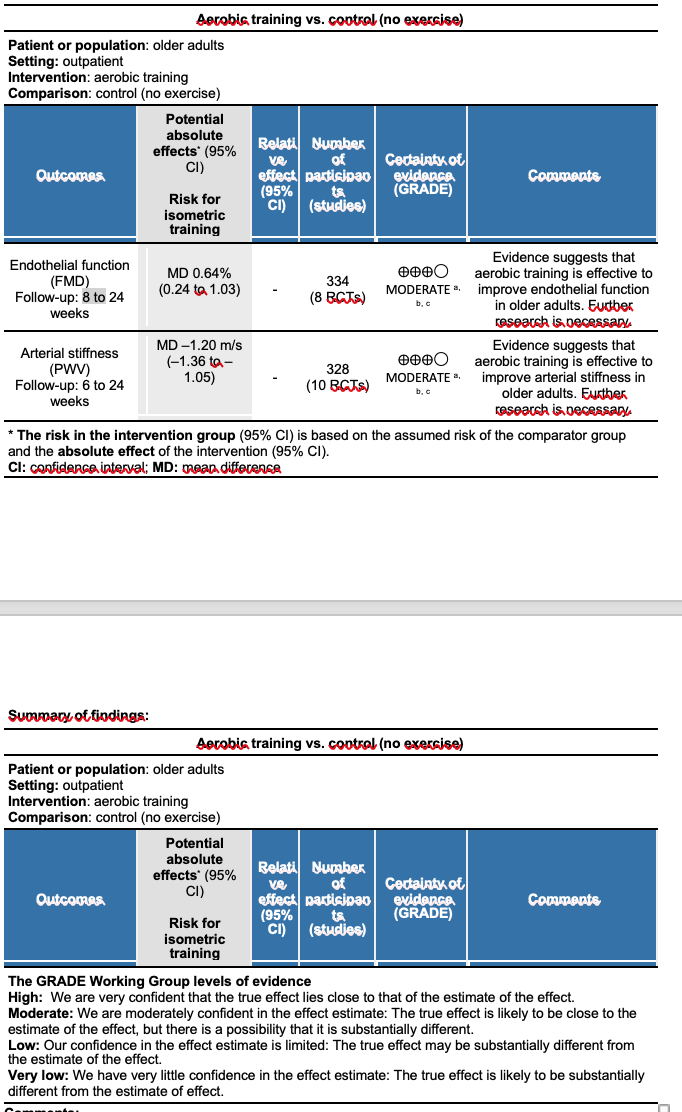

Supplement: S1 Fig — (TIFF) [file pone.0308600.s004.tiff]

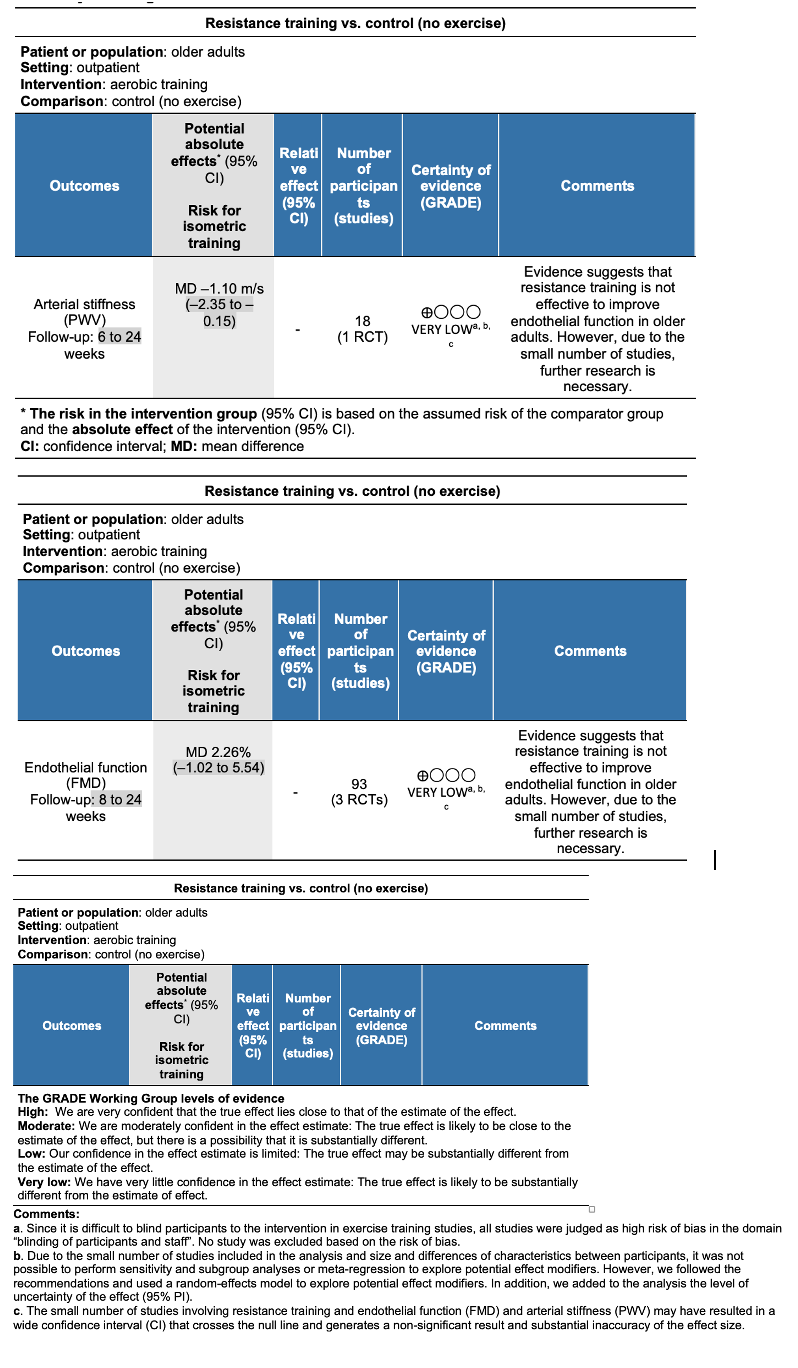

Supplement: S2 Fig — (TIFF) [file pone.0308600.s005.tiff]

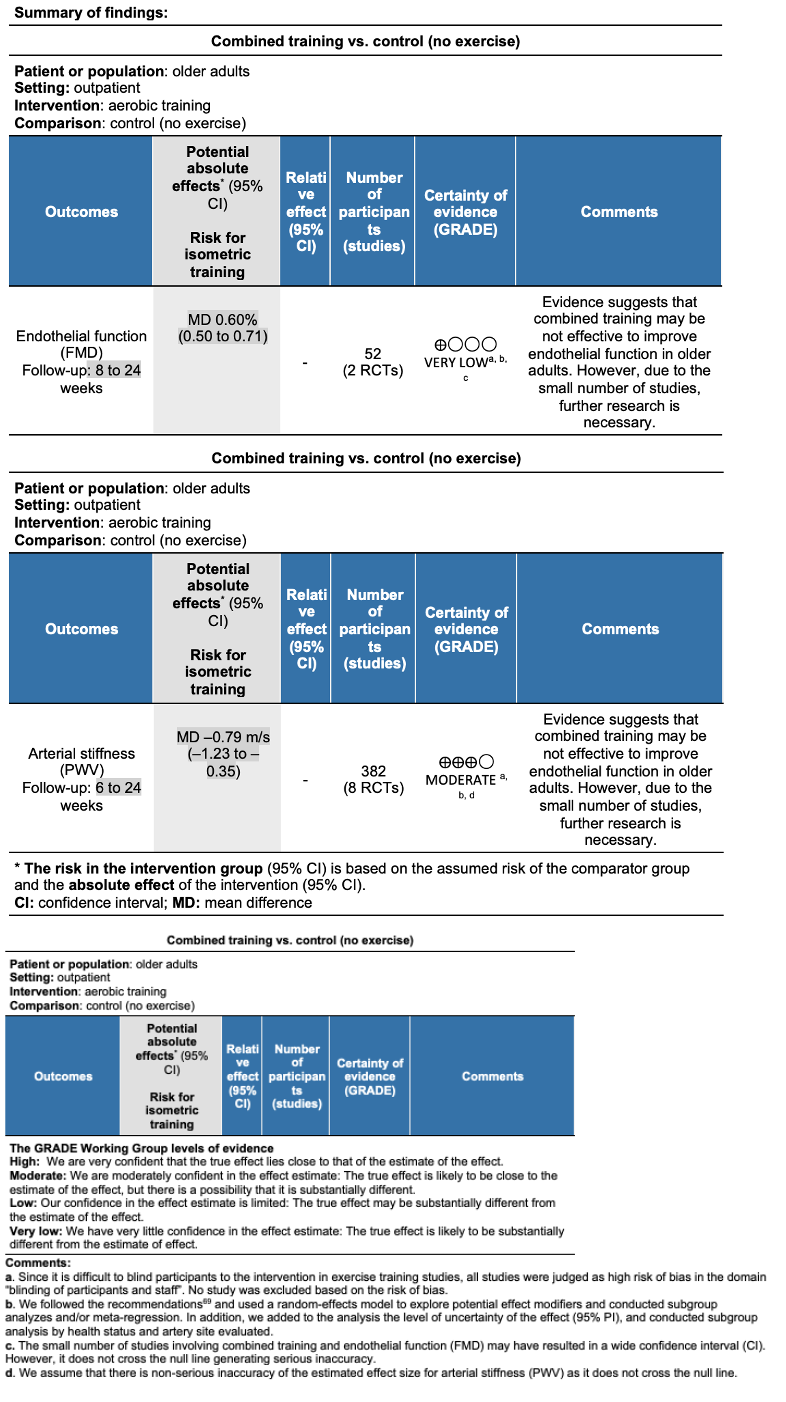

Supplement: S3 Fig — (TIFF) [file pone.0308600.s006.tiff]

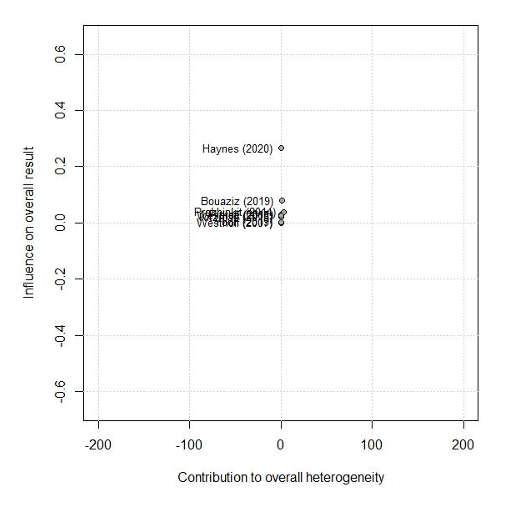

Supplement: S4 Fig — (TIFF) [file pone.0308600.s007.tiff]

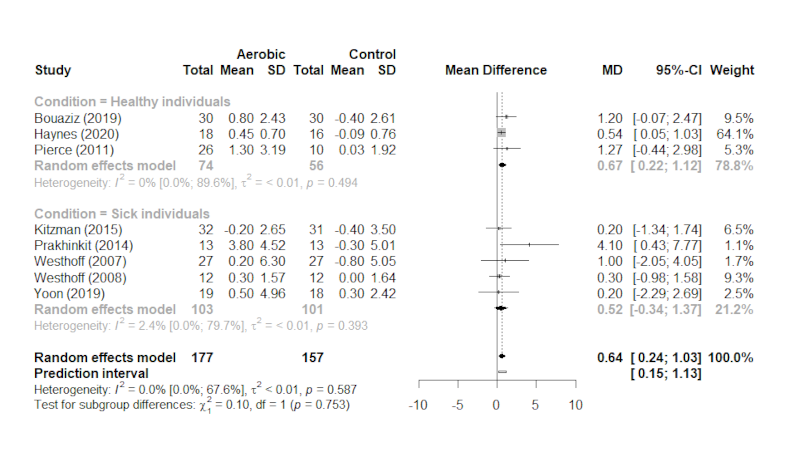

Supplement: S5 Fig — (TIFF) [file pone.0308600.s008.tiff]

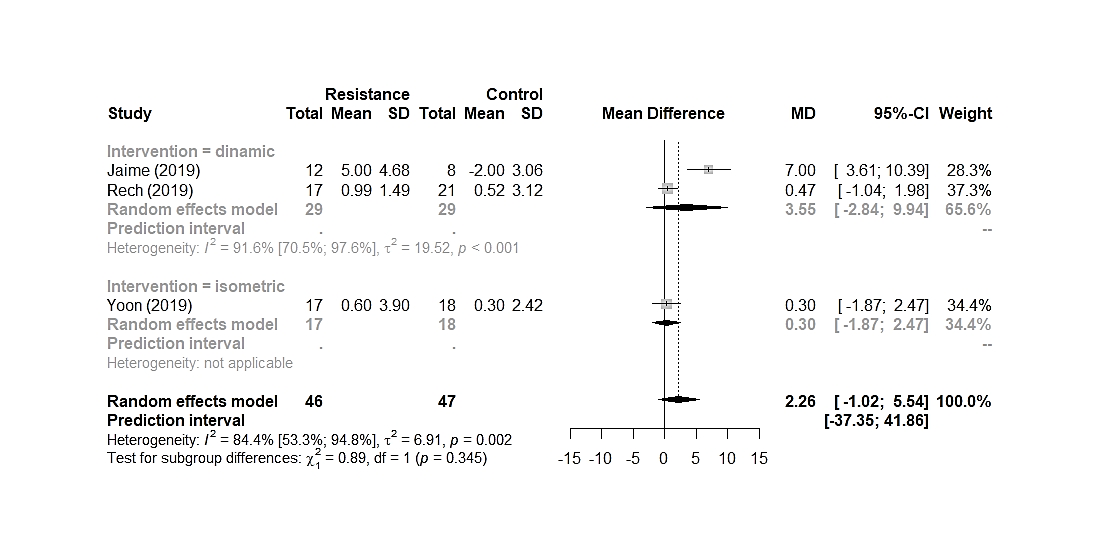

Supplement: S6 Fig — (TIFF) [file pone.0308600.s009.tiff]

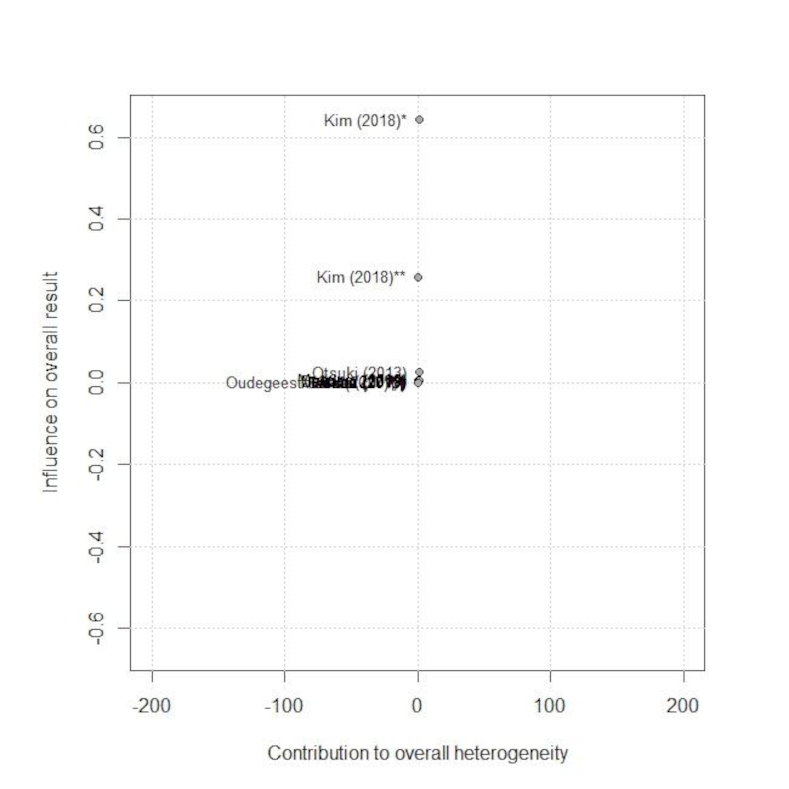

Supplement: S7 Fig — (TIFF) [file pone.0308600.s010.tiff]

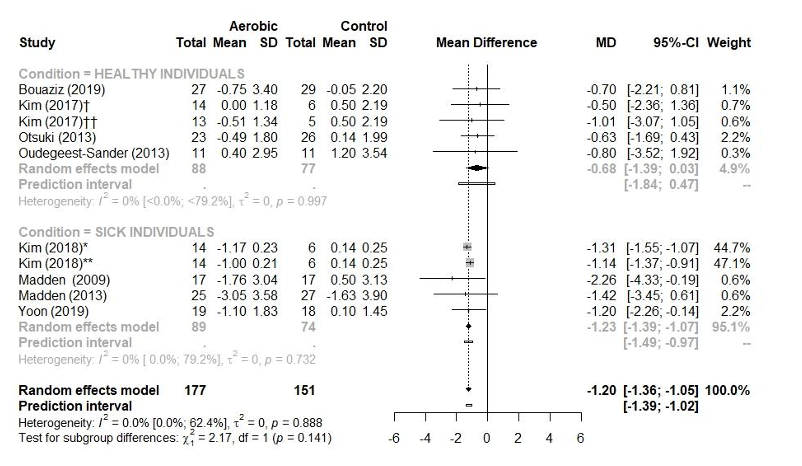

Supplement: S8 Fig — (TIFF) [file pone.0308600.s011.tiff]

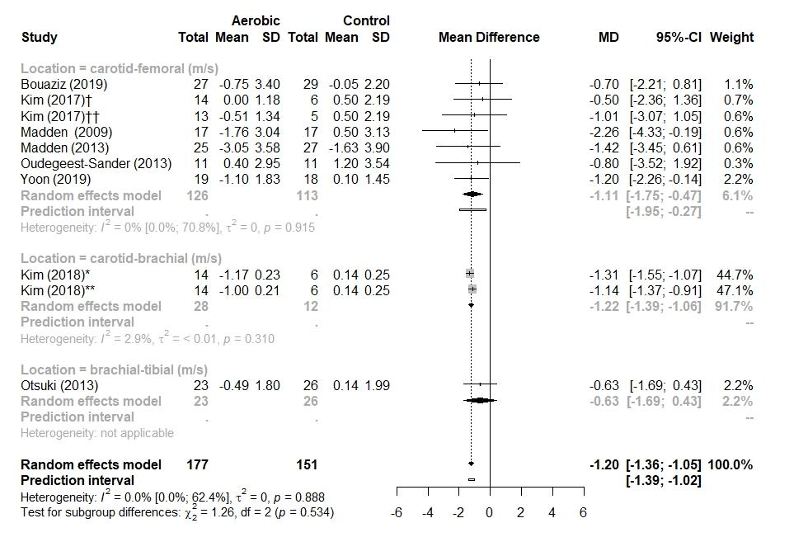

Supplement: S9 Fig — (TIFF) [file pone.0308600.s012.tiff]

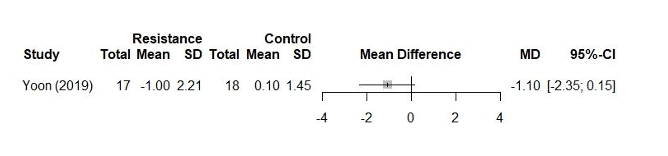

Supplement: S10 Fig — (TIFF) [file pone.0308600.s013.tiff]

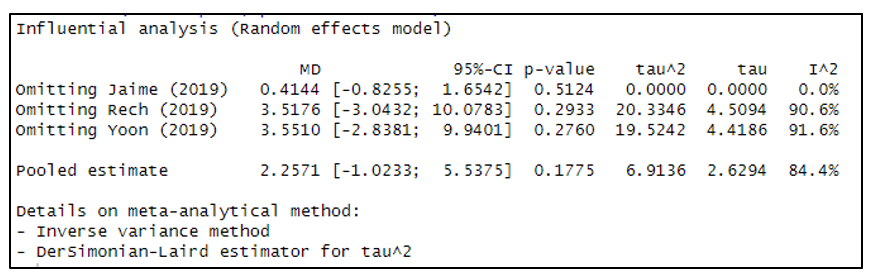

Supplement: S11 Fig — (TIFF) [file pone.0308600.s014.tiff]

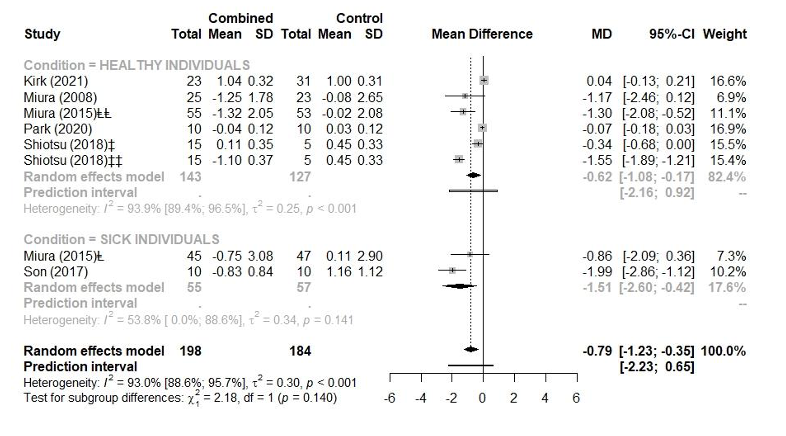

Supplement: S12 Fig — (TIFF) [file pone.0308600.s015.tiff]

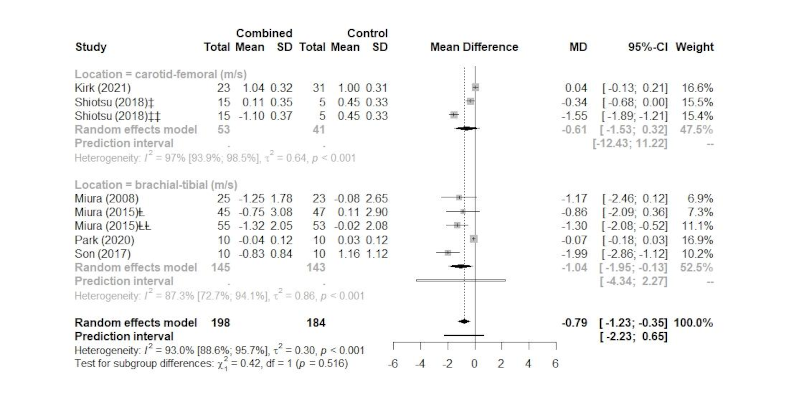

Supplement: S13 Fig — (TIFF) [file pone.0308600.s016.tiff]

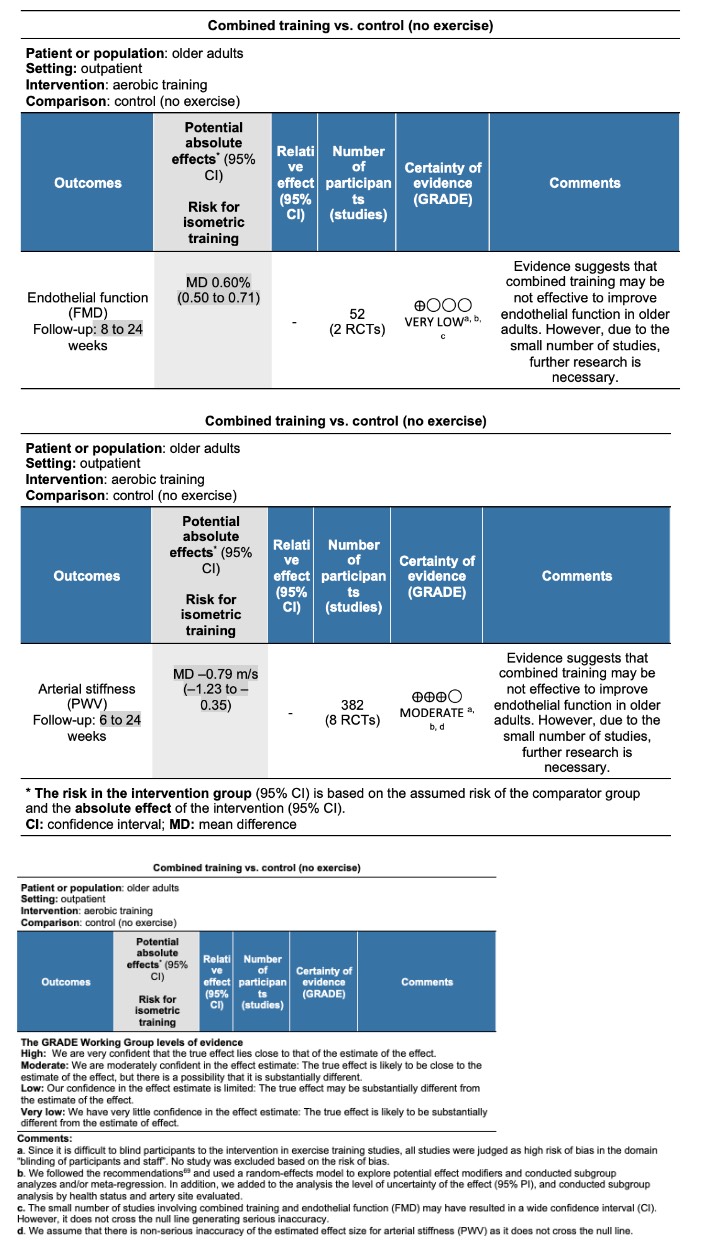

Supplement: S15 Fig — (JPEG) [file pone.0308600.s018.jpeg]
